# Modeling Sarcoglycanopathy in *Danio rerio*

**DOI:** 10.3390/ijms241612707

**Published:** 2023-08-11

**Authors:** Francesco Dalla Barba, Michela Soardi, Leila Mouhib, Giovanni Risato, Eylem Emek Akyürek, Tyrone Lucon-Xiccato, Martina Scano, Alberto Benetollo, Roberta Sacchetto, Isabelle Richard, Francesco Argenton, Cristiano Bertolucci, Marcello Carotti, Dorianna Sandonà

**Affiliations:** 1 Department of Biomedical Sciences, University of Padova, Via U. Bassi 58/b, 35131 Padova, Italy; francesco.dallabarba@unipd.it (F.D.B.);; 2 Randall Center for Cell and Molecular Biophysics, King’s College London, London WC2R 2LS, UK; 3 Department of Biology, University of Padova, Via U. Bassi 58/b, 35131 Padova, Italy; 4 Department of Cardiac-Thoracic-Vascular Sciences and Public Health, University of Padova, Via Giustiniani, 2, 35128 Padova, Italy; 5 Department of Comparative Biomedicine and Food Science, University of Padova, Agripolis, Legnaro, 35020 Padova, Italy; 6 Department of Life Sciences and Biotechnology, University of Ferrara, Via Luigi Borsari 46, 44121 Ferrara, Italy; 7 Genethon, F-91002 Evry, France; 8 INSERM, U951, INTEGRARE Research Unit, F-91002 Evry, France

**Keywords:** limb girdle muscular dystrophies, δ-sarcoglycan, β-sarcoglycan, animal models, genome editing, knockout

## Abstract

Sarcoglycanopathies, also known as limb girdle muscular dystrophy 3-6, are rare muscular dystrophies characterized, although heterogeneous, by high disability, with patients often wheelchair-bound by late adolescence and frequently developing respiratory and cardiac problems. These diseases are currently incurable, emphasizing the importance of effective treatment strategies and the necessity of animal models for drug screening and therapeutic verification. Using the CRISPR/Cas9 genome editing technique, we generated and characterized δ-sarcoglycan and β-sarcoglycan knockout zebrafish lines, which presented a progressive disease phenotype that worsened from a mild larval stage to distinct myopathic features in adulthood. By subjecting the knockout larvae to a viscous swimming medium, we were able to anticipate disease onset. The δ-SG knockout line was further exploited to demonstrate that a δ-SG missense mutant is a substrate for endoplasmic reticulum-associated degradation (ERAD), indicating premature degradation due to protein folding defects. In conclusion, our study underscores the utility of zebrafish in modeling sarcoglycanopathies through either gene knockout or future knock-in techniques. These novel zebrafish lines will not only enhance our understanding of the disease’s pathogenic mechanisms, but will also serve as powerful tools for phenotype-based drug screening, ultimately contributing to the development of a cure for sarcoglycanopathies.

## 1. Introduction

Sarcoglycanopathy is the collective name of four rare autosomal recessive diseases characterized by the involvement of the proximal musculature of the upper and lower limb girdle. The disease is due to mutations in the *SGCA*, *SGCB*, *SGCG*, and *SGCD* genes that code for α-, β-, γ-, and δ-sarcoglycan (SG). Accordingly, the four forms of the disease are also referred to as limb girdle muscular dystrophy LGMDR3-6 [1,2]. SGs form a tetramer that is part of the dystrophin associated protein complex (DAPC) that plays a key role in protecting sarcolemma from stress deriving from muscle contraction [3,4]. The genetic defects responsible for sarcoglycanopathy are, in most cases, missense mutations, accounting approximately for 67% of all cases, followed by frame shift and null mutations [5]. Mutations in *SGC* genes result in the severe reduction/loss of the mutated protein and the secondary deficiency of the wild type partners, with null and out of frame mutations being the worst for complex biogenesis. This alters the DAPC properties and leads to progressive muscle degeneration [5]. Most cases of sarcoglycanopathy are severe and characterized by early onset and rapid progression with loss of ambulation during adolescence. Mild forms are also known, with late onset and slow progression and even nearly asymptomatic patients having been described [6,7]. Respiratory problems requiring ventilation support are common in sarcoglycanopathy. Dilated cardiomyopathy may develop in all forms, needing constant heart surveillance, especially for LGMDR4 and R6 patients [8,9,10]. Cognitive impairment has never been reported.

Several rodent models of sarcoglycanopathy are available, both naturally occurring and as the consequence of genome modification [11,12]. In mice, mutations disrupting a specific sarcoglycan gene result in phenotypic-features well-resembling the human condition. All these KO animals present signs of progressive dystrophy in skeletal musculature, whereas dilated or hypertrophic cardiomyopathy develops in β- γ-, and δ-SG-, but not in α-SG-null mice [13,14,15,16,17]. 

In the attempt to find a therapeutic solution for sarcoglycanopathies, which are still orphan diseases, great interest has been devoted to the search for alternative animal models of the disease, mirroring the phenotype, and at the same time, allowing for the in vivo screening of compounds, unthinkable in mice for both ethical, time, and cost issues. Among the vertebrates, *Danio rerio*, commonly known as zebrafish, is gaining increased interest as an animal model of human diseases and it is widely used in phenotype-based drug discovery experiments [18,19]. Here, we present data concerning the generation and characterization of novel mutated zebrafish lines in which CRISPR/Cas9 technology was applied to knockout the δ- and β-SG genes. We characterized the mutated zebrafish during the early stages of development and adulthood. Like in humans, the disease is progressive, with slight functional muscular impairment during the larval stage, while a severe muscular and cardiac phenotype develops in adulthood. To promote an early onset of the disease, we subjected zebrafish larvae to stressful conditions for skeletal muscle, eventually highlighting precocious muscle damage. Finally, to prove the suitability of zebrafish in modeling sarcoglycanopathy, we successfully proved the involvement of the endoplasmic reticulum associated degradation (ERAD) pathway in the fate of one δ-SG missense mutant. 

## 2. Results

### 2.1. Generation and First Characterization of the δ-SG and β-SG KO Zebrafish Lines

**Figure 1:**
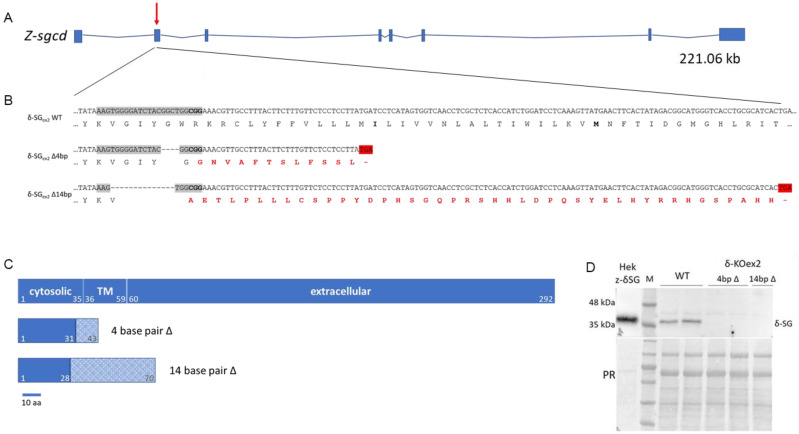
*Sgcd*^−/−^ zebrafish lines, mutations introduced, and consequences at the protein level. (**A**) Genomic organization of the wild type *z*-*sgcd* gene: boxes, exons; lines, introns. The red arrow points the site in exon 2 targeted by Cas9. (**B**) Nucleotide and amino acid sequences of the wild type and 4-bp and 14-bp deletion mutants (δ-SG_ex2_KO^Δ4^ and δ-SG_ex2_KO^Δ14^, respectively), as revealed by DNA sequencing analysis of the *sgcd* CRISPR target site. Each deleted nucleotide is represented by a dash, the CRISPR target site is highlighted in grey with the PAM sequence in bold. The amino acid sequence of the wild type (black amino acid) and of the mutants is reported under the nucleotide sequence. For both mutants, the consequence of the deletions is a frame shift (red amino acids) with the appearance of a premature stop codon (after 12 amino acids in the δ-SG_ex2_Δ4bp and 42 amino acids in the δ-SG_ex2_Δ14). (**C**) Scheme of the primary sequence of the wild type δ-SG protein and of the predicted truncated form with the different topological domains. ∆, deletion (**D**) Western blot (WB) analysis, showing the absence of the δ-SG protein in the lysates from embryos, 72 h post fertilization (hpf), of both *sgcd*^−/−^ zebrafish lines. Lysates from HEK293 expressing the zebrafish δ-SG sequence (Hek z-δSG) and from wild type zebrafish embryos were used as positive controls.

Orthologs of all human muscle-specific SGs are expressed in zebrafish [20]. Protein sequence alignment shows that among the four SG proteins, δ- and β-SG are the most conserved. The *z*-*sgcd* gene, localized on chromosome 21, is composed of eight exons and seven long introns, and codes for a protein of 292 amino acids (Figure 1A). The *z-sgcb* gene maps on chromosome 20; it is composed of six exons coding for a protein of 323 amino acids (Figure S1A). For gene knockout, we exploited the CRISPR/Cas9 technology targeting exon 2 in both genes. Figure 1B reports the region of exon 2 of the *sgcd* gene where the annealing site of the single guide RNA (sgRNA) and the protospacer adjacent motif (PAM) are highlighted. More than 300 single cell fertilized zebrafish oocytes were injected with the sgRNA (Table S1) and the CRISPR/Cas9 ribonucleoprotein complex. Larvae were raised to adulthood as a chimeric F0 population. The activity of Cas9, guided by the sgRNA, produced double strand breaks (DSB) at the specific genome position, which were repaired by the cells through non-homologous end joining (NHEJ). This pathway being a low fidelity repair process, the procedure resulted in the introduction of indel mutations [21].

At the end of the selection process, we isolated one clone presenting a deletion of 4 bp (clone δ-SG_ex2_Δ4) and one clone with a deletion of 14 bp (clone δ-SG_ex2_Δ14) in exon 2 of the *sgcd* gene (Figure 1B). In both cases, the mutation led to an out of frame δ-SG polypeptide with the appearance of premature stop codons (Figure 1C). The appearance of the premature stop codon is predicted to activate the nonsense-mediated decay of the mRNA. As expected, no protein signal was revealed by Western blot (WB) analysis of the protein lysates from larvae of the two mutated zebrafish lines (Figure 1D). From this point, we used the δ-SG_ex2_Δ4, named *sgcd*^−/−^, in all of the subsequent experiments. This choice was made to minimize any possible, although not expected, interference of the truncated polypeptides deriving from the out-of-frame sequences. Indeed, if translation were to occur, the product from δ-SG_ex2_Δ4 (43 amino acids) is much shorter than the one from δ-SG_ex2_Δ14 (70 amino acids).

The procedure adopted for knocking out the *sgcb* gene and the WB results are reported in the Supplementary Materials as Figure S1B–D.

The analysis of possible off-targets (Table S2) in the *sgcd^−/−^* line is reported in the Supplementary Materials. 

Despite the absence of either δ-SG or β-SG, no alteration in the fish morphology was observed during the first days of development that was adherent to the general morphological score [22,23]. At 72 hpf, the mean body length of the mutants was slightly, but statistically significantly shorter than their wild type siblings (Figure S2A), however, at 6 days post fertilization (dpf), the length was essentially identical (Figure S2B). The morphological analysis of the populations at 7 dpf evidenced the presence of some larvae with a curved spine, about 13% and 16.7% in δ-SG and β-SG KO, respectively, while an average of 4.1% of fish with altered morphology was observed in their wild type siblings (Figure S2C). 

From this point onward, we focused the attention on the *sgcd^−/−^* line since the *sgcb^−/−^* line showed problems of both low fertility and a variability of different matings in reaching adulthood. When possible, data regarding the *sgcb^−/−^* line have been reported anyway, mainly presented in the Supplementary Materials. 

When the survival rate of the *sgcd^−/−^* line was determined, it was clear that the mutated and wild type fish behaved similarly up to 10 dpf. Conversely, when fish started to feed autonomously, the curves diverged significantly, with an average of 40% of *sgcd^−/−^* fish reaching adulthood in comparison to about 60% of the wild type ones (Figure S3). 

On the whole, these findings indicate a milder phenotype of the null mutants, particularly at the early stage of development. This is rather different from what was observed in fish resulting from the knocking down of δ-SG with morpholino oligos (MO), in which a severe phenotype was evident since 24 hpf [24,25] (Supplementary Materials Figure S4).

### 2.2. Swimming Ability of sgcd^−/−^ and sgcb^−/−^ Larvae

**Figure 2:**
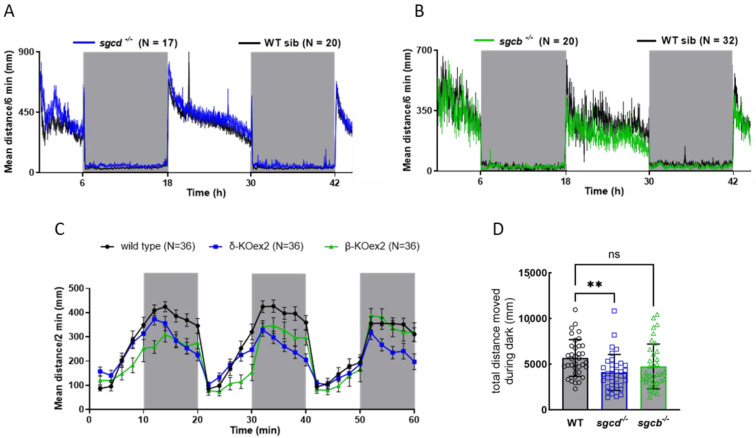
Swimming ability of the *sgcd*^−/−^ and *sgcb*^−/−^ larvae. (**A**) *sgcd^−/−^* and wild type sibling larvae, (**B**) *sgcb^−/−^* and wild type sibling larvae at 4 dpf were placed in a 48-well plate and introduced into the DanioVision tracking system. After a few hours of acclimation, the zebrafish larvae movements were recorded for 48 h under a 12:12 light–dark cycle; grey boxes represent the dark periods. The distance moved every 6 min by each larva was recorded and plotted for the entire period considered; each point is the mean distance covered by the number of larvae as indicated, standard deviation was omitted for clarity. (**C**) Startle test in which 5 dpf zebrafish larvae, upon 20 min of habituation, were subjected to three cycles of 10 min of light, followed by 10 min of dark (grey boxes). The graph reports the mean distance moved every 2 min ± SE of 36 larvae for each genotype. As expected, larvae activity increased during the dark periods, slowing down to a basal level during the light periods. (**D**) Quantification of the total distance moved during the three dark periods of the startle test by fish of the three genotypes; the average activity value ± SE is also indicated. Statistical analysis was performed using the one-way ANOVA test followed by Dunnett’s multiple comparisons test. ns, *p* > 0.05; **, *p* ≤ 0.01.

To evaluate the swimming performance of the novel mutated zebrafish lines, we exploited the DanioVision observation chamber, an instrument that allowed us to record the movement of individual zebrafish larvae placed into a multi-well plate. The temperature and photoperiod can be rigorously controlled, allowing the behavior of the embryos to be followed, both in physiological and stress conditions and at established time intervals [26,27,28]. First, we recorded the movement of wild type and mutant larvae in a 12:12 light–dark cycle for 48 h. Larvae were introduced into the chamber at 4 dpf and followed until the beginning of 6 dpf. Fish were almost stationary during the period of darkness, while they immediately activated when the light was switched on, then progressively reduced their swimming activity but remained active until the light was switched off again [29,30]. In Figure 2A,B, it is possible to observe that both the SG-null lines behaved similarly to those of the sibling wild type. This suggests that at this stage of development, the minimal morphological alterations observed had no detrimental effect on their free-swimming ability. Therefore, to disclose possible differences in muscle functionality, we challenged larvae with a stressful condition. For this purpose, we performed a light startle test in which zebrafish larvae were subjected to cycles of rapid changes in light intensity (three cycles of 10 min of light followed by 10 min of dark). The sudden reduction in light is perceived by zebrafish as a danger signal, inducing a rapid swimming increase to escape a potential *peril* [31,32]. In the presence of muscular weakening, the mutants are expected to swim a reduced distance during the dark periods compared to the wild type. In Figure 2C, the comparable overall behavior of the three genotypes with a slightly reduced distance moved during the dark period by the mutants is evident. This reduction, however, was statistically significant for the *sgcd^−/−^* zebrafish line only (Figure 2D). 

### 2.3. Analysis of Myofiber Integrity of sgcd^−/−^ Larvae

**Figure 3:**
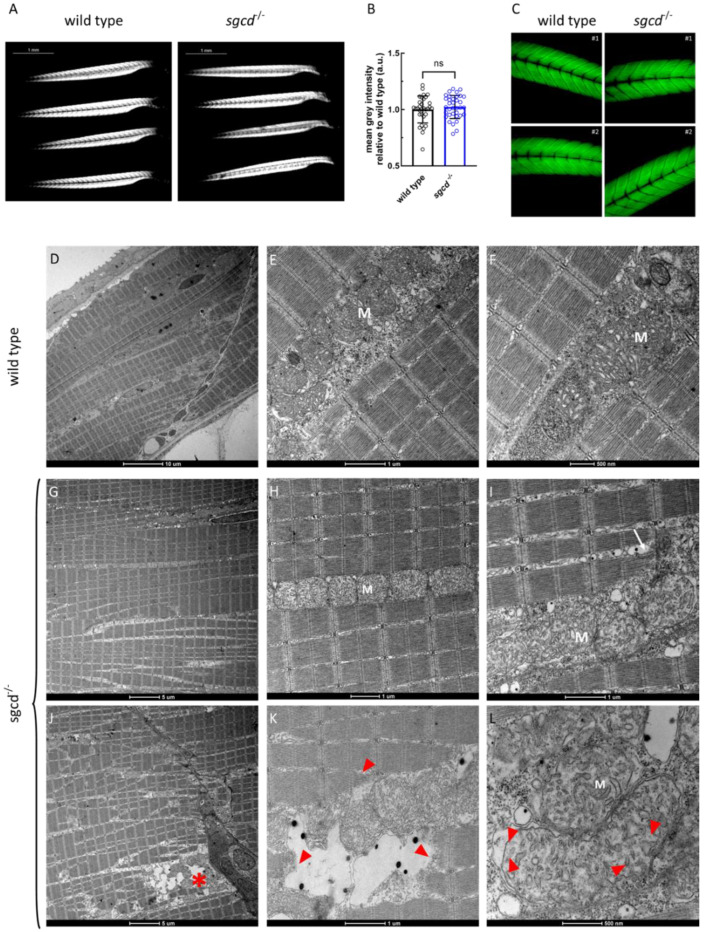
Muscle fiber integrity at the macroscopic and ultrastructural level in the *sgcd*^−/−^ and wild type larvae. (**A**) Representative birefringence images of the larvae of the two genotypes as obtained by viewing the zebrafish with a plane polarizing filter. Microscope’s magnification 2.5×. (**B**) Birefringence quantification. Statistical analysis was performed by the Mann–Whitney test; ns, *p* > 0.05. (**C**) Representative images of the phalloidin staining of whole zebrafish larvae at 5 dpf. Microscope’s magnification 20×. (**D**–**L**) Representative ultrathin sections from larvae at 5 dpf. Scale bar: um, µm. The well-preserved array of sarcomeres is evident in both the wild type (**D**–**F**) and mutated (**G**–**I**) larvae. In (**J**), this organization is partially lost, and some fibers are detached from the myosepta (asterisk in (**J**)), where myofibrillar disarrangement is evident (red arrowheads in (**K**)). Triad organization in *sgcd^−/−^* is almost indistinguishable from that of the wild type (compare (**E**) with (**H**)), even if, in a few ultrastructural sections, it is possible to observe that the SR cisternae appeared dilated (white arrow in (**I**)). Mitochondria (M) appeared normal in both the wild type (**E**,**F**) and mutant (**H**,**I**) larvae, however, where fibers were damaged, mitochondrial alterations were evident, with detachment of the outer mitochondrial membrane and expansion of the intermembrane space (red arrowheads in (**L**)).

Muscle integrity in zebrafish can be simply visualized by exploiting the optical phenomenon called birefringence [33]. In Figure 3A, the sarcomeres appeared brilliant and the signal was quite regularly distributed along the entire body in both the wild type and *sgcd^−/−^* lines at 5 dpf. In the larvae of the mutated zebrafish, it was possible to observe a few of the myotomes presenting a reduced signal that, however, was not statistically significant in comparison to the wild type (Figure 3B). These data were qualitatively confirmed by the phalloidin staining of the wild type and *sgcd^−/−^* larvae at 5 dpf (Figure 3C). 

Ultrastructural analysis, carried out by transmission electron microscopy (TEM) (Figure 3D–L), evidenced the regular array of sarcomeres, the preservation of the triad organization, and the presence of mitochondria with well-developed tubular cristae in muscle tissue ultrathin sections of both the wild type (Figure 3D–F) and *sgcd^−/−^* (Figure 3G–I) zebrafish at 5 dpf. On the other hand, in a few sections of the mutant larvae (Figure 3J–L), it was possible to appreciate the typical features of damaged myofibers such as altered sarcomeric organization, dilation of the sarcoplasmic reticulum cisternae, detachment from the myosepta, and the degeneration of myofibrils and mitochondria.

Altogether, these findings suggest that, even if not macroscopically evident, muscle damage is present since the beginning of development in the mutated fish, and it is their progressive accumulation that will eventually lead to the manifestation of the myopathic phenotype.

### 2.4. Skeletal Muscle Structure and Ultrastructure of Wild Type and sgcd^−/−^ Adult Fish

**Figure 4:**
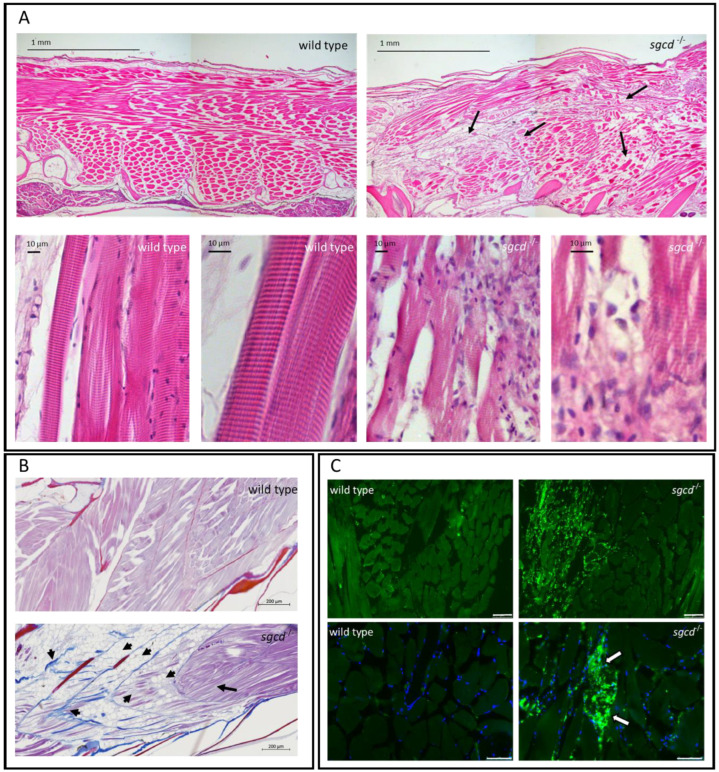
δ-SG deficiency causes defects in the skeletal muscle of adult zebrafish. (**A**) Hematoxylin and eosin staining of sagittal sections from adult wild type and *sgcd^−/−^* zebrafish. The skeletal muscle in the mutated animal was clearly affected, fibers (black arrows) were of different sizes and shapes, and appeared loosely interacting in comparison to the wild type. At high magnification, it is possible to observe that the classical sarcomeric striation, present in wild type tissue, was less evident in the mutant; both fibers and myofibrils were partially disorganized and wavy. Furthermore, a high number of mononucleated cells surrounded the damaged fibers. (**B**) Azan–Mallory staining of the sagittal section of the wild type and *sgcd^−/−^* zebrafish. Arrowheads indicate both the deposition of fibrotic tissue (colored in blue) and the presence of mature adipose tissue replacing the contractile one. The black arrow highlights the presence of a conserved region of skeletal muscle. (**C**) Immunofluorescence staining with L-plastin (green signal), a pan-leukocytic marker evidenced, at low magnification, the huge number of inflammatory cells that infiltrated the skeletal muscle tissue. Nuclei were counterstained with DAPI (blue signal). At higher magnification, it is possible to observe the L-plastin positive mononucleated cells surrounding a damaged skeletal muscle fiber (arrows). Upper panel bars correspond to 75 µm, lower panel bars to 50 µm Antibody against L-plastin is listed in Table S2.

Sarcoglycanopathies are progressive diseases, therefore, we analyzed one-year old fish from both the wild type and *sgcd^−/−^* genotype, fixed in PFA and subsequently embedded in paraffin. Ten µm thick sections were stained with hematoxylin and eosin (H&E). From the images reported in Figure 4A, it is possible to observe that the *sgcd^−/−^* fish presented a clear sign of myopathy in comparison to the wild type. Indeed, at low magnification, it is possible to appreciate the disorganization of the muscle fibers, which appeared loosely interacting and presented a variable diameter. 

At higher magnification (Figure 4A, lower), it was possible to observe the myofibrillar striation and the tight interaction among fibers in the wild type. On the contrary, the δ-SG KO fish muscle fibers were thinner and less compact. Furthermore, while the myofibrillar striation was partially preserved, it was possible to find regions with a high number of mononucleated cells surrounding and probably engaged in the removal of damaged fibers, highlighting sites of muscle degeneration and inflammation. Furthermore, it is evident that in the *sgcd^−/−^* animals, fibrotic and adipose tissue replaced the contractile one (Figure 4B). Skeletal muscle inflammation in the mutated zebrafish was further confirmed by immunofluorescence staining with the pan-leukocytic marker L-plastin (Figure 4C). While the signal from this marker was virtually absent in the adult wild type zebrafish, in the *sgcd^−/−^* fish, it was evident that the mononucleated cells surrounding myofibers, highly positive for L-plastin, were inflammatory cells.

**Figure 5:**
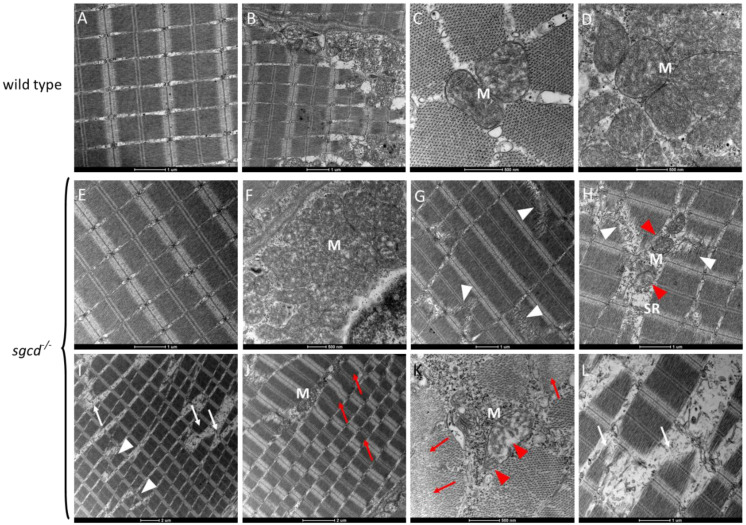
Transmission electron microscopy analysis of the skeletal muscle of the WT and *sgcd^−/−^* adult zebrafish lines. Ultrathin sections from 1-year-old WT zebrafish (**A**–**D**) and age-matched *sgcd^−/−^* zebrafish (**E**–**L**). Scale bar: um, µm. It is possible to observe that in some regions, the contractile tissue and mitochondria (M) of the mutant fish (panel (**E**,**F**)) were almost indistinguishable from the wild type (panels (**A**–**D**)). On the other hand, in most of the sections of *sgcd^−/−^* (panels (**G**–**L**)), clear signs of myopathy were present such as myofibrillar fragmentation (white arrowheads), fiber damage (white arrows), out-of-frame sarcomeric array (panel (**J**)), hypercontracted fibers (red arrows), and dilated sarcoplasmic reticulum (SR). In these regions, mitochondria are degenerating (panels (**H**,**K**), red arrowheads).

Adult *sgcd^−/−^* zebrafish ultrastructural analysis showed abnormalities, which correlated with the histological findings of the contractile tissue. Even though some areas of the contractile tissue seemed indistinguishable from the wild type one (compare Figure 5A with Figure 5E), in general, it was possible to observe muscle fibers displaying myofibrillar fragmentation (white arrowheads Figure 5G,I) in the ultrathin sections, an area of damaged and degenerating myofibers (white arrows in Figure 5I,L), and hypercontracted myofibrils (red arrows in Figure 5J,K) with out-of-frame organization of the sarcomeric arrays (Figure 5J). In ultrathin sections of the adult *sgcd^−/−^* muscle, the mitochondria varied from a normal morphology indistinguishable from the wild type (compare Figure 5F to Figure 5C or Figure 5D) to a slightly altered appearance (the cristae began to swell in Figure 5H red arrowheads) to an evident damaged structure (Figure 5K, red arrowheads). These features were most evident in regions with initial rupture or extensive myofiber degeneration.

These ultrastructural findings, together with the histological data, are suggestive of a clear and severe myopathic status developed by the *sgcd^−/−^* zebrafish line in adulthood, well-resembling the skeletal muscle phenotype of MDs like sarcoglycanopathies [34,35,36]. Similar results were observed in ultrathin sections of *sgcb^−/−^,* as reported in Supplementary Materials Figure S5.

### 2.5. Swimming Performance of Adult sgcd^−/−^

**Figure 6:**
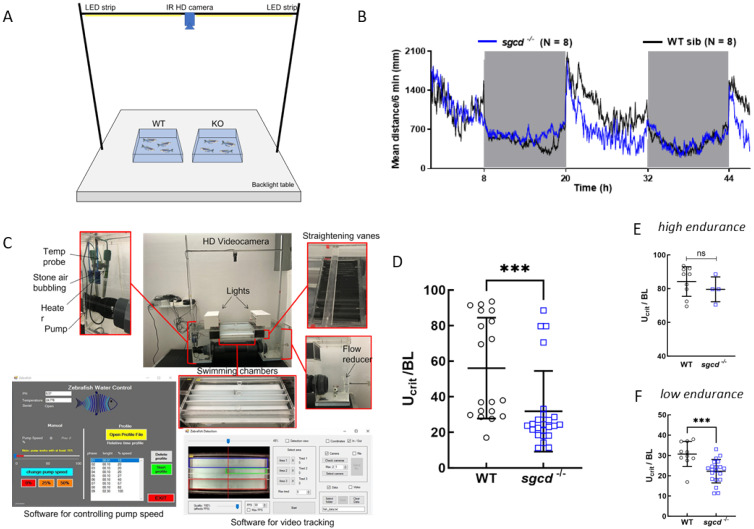
Swimming performance of the adult wild type and *sgcd*^−/−^ zebrafish. (**A**) Tracking system and representative arena of 40 × 40 cm for adult zebrafish. (**B**) Distance moved by 7-month-old *sgcd*^−/−^ and wild type siblings. After a few hours of habituation, the fish movements were recorded for 48 h under a 12:12 LD cycle. Each point represents the mean value of the distance moved in 6 min by eight wild type (black trace) and eight mutated animals (blue trace). With this set-up, it is not possible to record the movement of a single fish. (**C**) The in-house developed swimming tunnel system that was conceived to allow for the simultaneous testing of three adult zebrafish in the swimming chambers (Lucon-Xiccato et al., 2021 [37]). (**D**) Swimming performance of adult fish (1-year-old), 19 wild type and 24 *sgcd*^−/−^. Fish were introduced in the swimming chambers and allowed to habituate for 60 min. Then, a counter current speed of 81.6 (cm/s) was applied. Fatigue was evaluated by measuring the time at which the fish stopped swimming counter current and contacted the rear section of the tunnel for >5 s (U_crit_). This value was normalized by the length of the fish. It is possible to see that the *sgcd*^−/−^ fish were less resistant than the wild type ones. Two subpopulations, high endurance (**E**) and low endurance (**F**) fish, were distinguishable in both genotypes. Statistical analysis was performed by the Mann–Whitney test; ns, *p* > 0.05; ***, *p* ≤ 0.001.

To evaluate the skeletal muscle functionality of adult *sgcd^−/−^* zebrafish, we recorded the swimming behavior at the basal condition (Figure 6A) of the seven-month old δ-SG-KO and wild type and measured the distance moved in a total period of 48 h. *Sgcd*^−/−^ fish behaved similarly to the wild type during the dark phases (Figure 6B). When the light was switched on, the swimming activity increased in both groups as expected [29]. In the subsequent hours, the wild type fish first reduced, then stabilized their activity, which was maintained along the residual light period. Conversely, the *sgcd*^−/−^ fish rapidly and progressively decreased their swimming to levels comparable to those of the dark phase (Figure 6B), suggesting muscle weakness.

To better assess the swimming performance of adult *sgcd^−/−^*, we challenged zebrafish in stressful conditions (counter current swimming) using an in-house built swimming tunnel (Figure 6C) [37]. The swimming performance was evaluated by measuring the fatigue of the animals as the time at which the fish stopped swimming counter-current and contacted the rear section of the tunnel for >5 s (U_crit_) [38]. After testing, the fish body dimensions were measured, and it was possible to observe that one-year-old *sgcd^−/−^* fish (both males and females chosen randomly) presented a body length and fin length similar to the wild type (Figure S6A,B) while their major body thickness was substantially lower (Figure S6C). In Figure 6D, it is interesting to observe that the mutated fish presented a U_crit_ significantly lower than the wild type. This was expected considering the presence of clear myopathic characteristics (Section 2.3 and Section 2.4), which evidently affected the strength and fatigability of the fish. Furthermore, it is possible to appreciate the presence of two populations in both genotypes that we classified as *high endurance fish* and *low endurance fish*. This finding resembles that already observed in the work by Lucon-Xiccato et al. [37] and can be explained by inter-individual differences of the original wild type strain, possibly due to unpredictable epigenetic modifications. In particular, the *low endurance population* of *sgcd*^−/−^ performed significantly worse than the wild type (Figure 6F). Conversely, in the *high endurance population*, the swimming activity of *sgcd*^−/−^ fish, even if reduced, was not statistically significantly different in comparison to the wild type (Figure 6E). This latter analysis, however, may have been affected by the small size of the two populations.

### 2.6. Heart Phenotype of the Adult sgcd^−/−^ Zebrafish

Sarcoglycanopathy, particularly LGMDR4 and LGMDR6, are characterized by progressive skeletal and cardiac impairment. Figure S7A reports the images of two representative hearts explanted from 1-year-old wild type and *sgcd^−/−^* fish. Since the first observation, it appears that the heart of the mutated zebrafish was roundish in comparison to the classical conical shape of the wild type one. The H&E staining (Figure S7B), performed on sagittal sections from whole fixed animals, revealed a less dense structure of the *sgcd^−/−^* heart ventricle in comparison to the wild type, with thinning of the ventricular wall. At high magnification, it is possible to observe that in the wild type heart, both the compact myocardium and the trabecular myocardium were homogeneous and well-organized [39,40]. Conversely, in the *sgcd^−/−^* heart, the compact myocardium appeared quite heterogenous, with certain regions composed of no more than one/two cell layers; similarly, the trabecular myocardium was thinner and markedly deranged. These findings are indicative of cardiomyopathy in the adult mutated zebrafish line, in this case also mimicking a condition observed in LGMDR6 and other MD subjects [9,41]. Further experiments will dissect the onset and progression of the cardiac disease by analyzing *sgcd^−/−^* hearts at different ages.

### 2.7. Skeletal Muscle Structure of Wild Type and sgcd^−/−^ Larvae upon Growth in Stressful Condition

**Figure 7:**
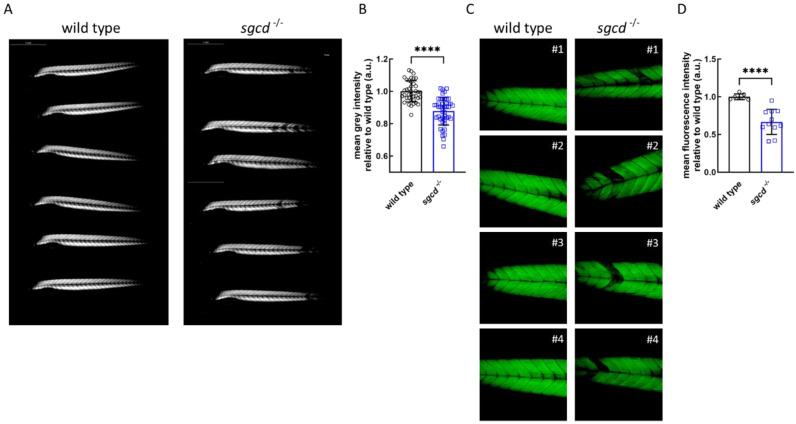
Muscle fiber integrity in the representative wild type and *sgcd*^−/−^ larvae growing in fish water with 1% methylcellulose added. (**A**) Representative birefringence images as obtained by viewing zebrafish with a plane polarizing filter. Microscope’s magnification 2.5X. (**B**) Birefringence quantification of zebrafish larvae as in (**A**) (wild type N = 39; *sgcd*^−/−^ N = 45). (**C**) Representative pictures of the phalloidin staining of whole zebrafish larvae at 5 dpf. Microscope’s magnification 20X. (**D**) Quantification of the fluorescence intensity of zebrafish larvae stained with phalloidin as in (**B**) (wild type N = 7; *sgcd*^−/−^ N = 11). Statistical analysis was performed by the Mann–Whitney test; ****, *p* ≤ 0.0001.

Our data suggest that the lack of one SG has a limited impact on zebrafish musculature during the first stages of development and that it is necessary to wait until adulthood to observe a clear muscular and cardiac phenotype. Therefore, with the scope of inducing the rise of dystrophic features early in development, we decided to grow *sgcd*^−/−^ and control wild type zebrafish in fish water with the addition of 1% methyl cellulose from 2 to 5 dpf [42]. In mammals, it has been clearly described that altered organization of the DAPC observed in sarcoglycanopathy reflects on the stability and integrity of the sarcolemma [4,5]. Thus, if the sarcolemma of the SG-null zebrafish is more fragile than that of the wild type, swimming in harsh conditions (viscous medium) is expected to produce skeletal muscle damage because a greater force is needed to move. In Figure 7A, it is possible to observe that the birefringence pattern of the mutants was substantially diverse from the wild type larvae, with a patchy signal from myotomes, a sign of damaged sarcomeres, primarily located in the back of the trunk and tail. Phalloidin staining was coherent with these findings, and in Figure 7C, it is clear the disorganization of the muscle structure in comparison to the wild type, with several myotomes presenting waving, less compacted fibers, some detached from the myosepta. The quantification of birefringence (Figure 7B) and phalloidin staining (Figure 7D) highlights the increased damage present at the skeletal muscle level. 

### 2.8. The E262K Missense Mutant of δ-SG Is Substrate of the ERAD Pathway in Zebrafish

**Figure 8:**
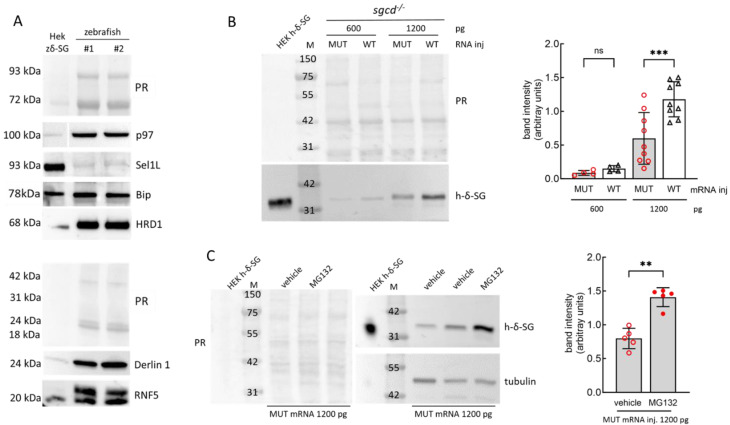
The E262K mutant of δ-SG is the substrate of the ERAD in zebrafish. (**A**) Western blot analysis of the expression of different components of the endoplasmic reticulum associated degradation (ERAD) in zebrafish. Lysates from 5 dpf larvae of the wild type zebrafish were resolved in SDS-PAGE and probed with antibodies specific for the indicated proteins (representative WBs are reported). The antibodies used are listed in Table S2; M, molecular markers; PR, Ponceau red staining used for protein content normalization. (**B**) *sgcd*^−/−^ zebrafish oocytes were microinjected with two concentrations (as indicated) of the human mRNA encoding either the wild type or mutated E262K-δ-SG. Embryos at 48 hpf were lysed and the total proteins purified. A representative WB (**left**), and densitometric quantification of human-δ-SG bands (**right**) are shown. The graph reports the band intensity values of the E262K-δ-SG (MUT) and wild type (WT) forms of the human protein at different concentrations of injected mRNA. The mean values ± SD from at least four independent microinjection experiments are reported. Statistical analysis was performed by one-way ANOVA followed by Šídák’s multiple comparisons test; ns, *p* > 0.05; ***, *p* ≤ 0.001. (**C**) *sgcd*^−/−^ zebrafish oocytes, at one cell stage, were microinjected with 1200 pg of the human E262K-δ-SG mRNA. At 24 hpf, embryos were treated with DMSO 1‰ (vehicle) or MG132 (10 µM). At 56 hpf, the embryos were lysed and the total proteins purified. A representative WB (**left**) and the densitometric quantification of human-δ-SG bands (**right**) are shown. The graph reports the band intensity values of the mutated protein in the two groups of embryos. The mean values ± SD from five independent microinjection experiments are reported. PR, Ponceau red staining. Blots were probed with rabbit polyclonal antibodies specifically recognizing the human δ-SG or tubulin, used for protein content normalization. Proteins purified from the HEK293 cells transfected with the human δ-SG sequence are reported as the positive control. Statistical analysis was performed by the Mann–Whitney test; **, *p* ≤ 0.01.

The nature of ERAD substrates has been verified for many SG missense mutants in mammalian cells. SGs undergo maturation and folding into the endoplasmic reticulum (ER). When a mutated sarcoglycan is unable to gain the native conformation, it is recognized as folding-defective and delivered to the ER exit sites. During the retro-translocation, it is ubiquitinated and eventually degraded by the proteasome in the cytosol [43,44,45,46]. Thus, with the main aim of verifying the suitability of zebrafish also as a model of sarcoglycanopathy when a SG carries a missense mutation, we microinjected *sgcd^−/−^* oocytes with the mRNA encoding the human E262K-δ-SG. If the amino acid substitution results in a folding defective δ-SG, the mutant protein should also be rapidly degraded in zebrafish. First, we verified the expression of many ERAD components. In Figure 8A, it is possible to observe that *D. rerio* expresses E3 ligases (HRD1 and RNF5), AAA-ATPase (p97 or VCP), chaperones (Bip), and adaptor proteins (Sel1L and Derlin). Noteworthy, most of these proteins have been identified in human cells as the ERAD elements responsible for the degradation of the α-SG mutant V247M [45].

Subsequently, we microinjected increasing amounts of either the wild type or human E262K-δ-SG mRNA in fertilized *sgcd^−/−^* oocytes. Two days post injection, the content of the human protein paralleled the amount of the mRNA injected (Figure 8B, WB and quantification). As expected, the densitometric analysis reported a lower content of the E262K-δ-SG protein in comparison to the wild type, with the best condition achieved by the injection of 1200 pg of mRNA. This suggests that the mutant carrying the amino acid substitution is a real ERAD substrate in zebrafish like in mammals. Afterward, we treated the embryos injected with the human E262K-δ-SG mRNA with the proteasome inhibitor MG132 for 32 h [47]. By inhibiting the ERAD executor, the δ-SG mutant content increased (Figure 8C), further signifying that the protein follows the same degradative pathway described in mammals, highlighting the zebrafish as a possible valid model for the forms of sarcoglycanopathy due to missense mutations [43,44,45,46]. 

## 3. Discussion

Sarcoglycanopathies are a group of rare muscular diseases presently without a cure [6]. They are caused by defects ranging from large to small deletions or insertions, null and missense mutations affecting sarcoglycan coding genes [5]. Sarcoglycans form a tetramer that, embedded in the dystrophin associated protein complex, plays a key role in protecting the muscle fibers’ membrane during muscle contraction. Sarcoglycanopathies are characterized by the loss/strong reduction in the mutated SG and the secondary deficiency of the wild type partners. With the disruption of the SG-complex, it is thought that, like in DMD, the sarcolemma becomes fragile and leaky. This results in the subsequent alteration in the intracellular calcium homeostasis, which can eventually lead to mitochondrial dysfunction. Thus, muscle is damaged during the normal contractile activity and undergoes progressive wasting [4,48,49]. The age of onset and the severity of the disease are inversely related to the residual level of the SGs at the sarcolemma. This heterogeneity may be linked to the type of mutation involved, with the phenotype resulting from large deletions or null mutations being the most severe [7]. Most cases of sarcoglycanopathy are characterized by cardiac impairment and respiratory insufficiency [9,10]. Much effort is still devoted to understanding the molecular pathogenesis of these diseases as this knowledge is of the utmost importance in designing novel therapeutic approaches. To this end, the availability of animal models mimicking the disease with high fidelity is crucial. Currently, the animal models that best mimic sarcoglycanopathies are SG-KO mice and the naturally occurring δ-SG mutants identified in several hamster strains. They develop muscle and heart problems, as in humans [11,50,51,52]. These mammals are of great help in proof-of-concept experiments and in the validation process of a novel therapeutic approach. However, they are unsuitable for in vivo phenotype-based drug screening for both financial and ethical concerns. For the same reasons, the investigation of the first steps of the pathogenic mechanism may also be difficult. Finally, it is important to mention that most of the genetic defects in sarcoglycanopathy are missense mutations, thus the proper model in these cases would be the knock-in (KI) animal. Even though the procedure to generate KI animals has been greatly improved by genome editing techniques, it is still a time-consuming and costly task in mammals. Furthermore, we must consider that the three SG-KI mice, generated until now, failed to develop a myopathic phenotype [44,53,54]. 

All of this considered, to model sarcoglycanopathy, we exploited the small vertebrate fish *Danio rerio* as more than 70% of human proteins (and about 82% of disease-causing human proteins) have an ortholog in zebrafish [55]. We generated two novel zebrafish lines in which either the δ-SG or β-SG had been knocked out. We focused on δ-SG and β-SG as the former (coded by *sgcd* gene) is 72% identical and 87% similar to the human ortholog while the second (coded by *sgcb* gene) is 75% identical and 87% similar to the human β-SG. Furthermore, published data [24,25] as well as our experiments of the transient downregulation of δ-SG expression by MO evidenced the role of such proteins in the development of a dystrophic phenotype, suggesting that zebrafish could mirror the sarcoglycanopathy phenotype. Taking advantage of the genome-editing approaches extensively applied in zebrafish [21,56], we inactivated the *sgcd* and the *sgcb* genes by using CRISPR/Cas9 technology, which resulted in the introduction of indel mutations at the level of the second exon in the two genes of interest. The final outcome in both cases was an out of frame sequence with the appearance of a precocious termination codon; no signal of the δ-SG or β-SG protein was revealed by WB. 

Despite our preliminary knockdown experiments using MO and in contrast to the published data [24,25], we surprisingly observed a very mild muscular phenotype at the early stage of development, even with the complete abolition of SG protein expression. In spite of the slight reduction in length soon after hatching, most mutant larvae were indistinguishable from the wild type at 6 dpf in both length and morphology. Furthermore, up to 10 dpf, the mortality rate of the mutants and wild type fish was very low and comparable. After this period, the survival rate of the *sgcd*^−/−^ decreased to stabilize around 40% of the initial embryos in comparison to the 60% of the wild type. This is surely related to the progressive nature of the disease as the muscular damage needs to accumulate to become phenotypically manifest, reducing the fitness of the fish. On the other hand, some precocious signs of muscular problems were present since the first step of development. For example, at 6 dpf, a small number of *sgcd*^−/−^ larvae presented a curved spine, which is a first sign of a myopathic phenotype in zebrafish. Furthermore, even though the SG knocked-out zebrafish lines showed unchanged swimming ability at resting conditions, a reduction in the locomotion performance was observed when the *sgdc*^−/−^ larvae were challenged with a startle test [57]. This is indicative of a first, even though mild, impairment of muscle function. All of these findings were confirmed by the analysis of the sarcomere birefringence properties and by phalloidin staining of the *sgcd*^−/−^ line. In comparison to the aged matched (5 dpf) wild type larvae, no statistically significant difference was observed, even though in mutants, a few of the myotomes appeared less brilliant. As expected on the basis of the above described analyses, the 5 dpf *sgcd*^−/−^ muscle at the ultrastructural level was practically indistinguishable from the control, showing well-preserved arrays of sarcomeres, conserved organization of the triads, and normal mitochondria. However, in a few images, it was possible to observe sites of muscle damage, where fibers were detached from the myosepta, with deranged myofilaments undergoing degeneration. At the same sites, the mitochondria appeared altered with outer membrane dilations and disrupted cristae, features reported in other zebrafish models of muscular dystrophies [49,58]. No cardiac abnormality was observed in the *sgcd^−/−^* zebrafish during the first days of development. 

In comparison with the novel SG-KO zebrafish, the phenotype of the morphants was much more severe, with more than 70% of individuals showing altered morphology, reduced/impaired motility, and a general high rate of mortality, resulting in an average of only 24% of the fertilized oocyte reaching 5 dpf [24,25] (Figure S3). The different outcomes could be partially due to some toxicity by the MO concentration, possible off-target activity of the MOs, genetic compensation mechanisms present in the mutants, or to other unforeseen reasons [59,60,61]. Thus, considering all of the above, we believe that it is important to carefully evaluate the outcome of the MO knocking down experiments in zebrafish because the alteration of a trait could sometimes be overestimated, which would also generate misleading conclusions. Nonetheless, one would be more confident of a conventional gene knockout outcome, which is expected to be more physio-pathologically consistent than transient animal manipulation.

As sarcoglycanopathies are progressive degenerative diseases [5,7], to have the whole characterization of the novel zebrafish lines, we evaluated the skeletal and cardiac muscle in adult fish. In these animals, we observed clear signs of dystrophy and cardiomyopathy, well-resembling the human condition [4,5,6,7]. The swimming performance of the *sgcd*^−/−^ fish was impaired in comparison to the wild type. This was observed at first by recording free fish swimming for more than 24 h. In this condition, the initial increase in movement of both genotypes in response to the switching on of the light [29,62] was followed by a rapid decline in the swimming activity in the mutants only, suggesting that these animals performed less than the controls. The evidence that the *sgcd*^−/−^ line suffered a higher fatigability was confirmed by the challenging experiment in the swim tunnel [37], where the *sgcd*^−/−^ fish, forced to swim countercurrent, stopped swimming significantly before the wild type. These data suggest a severe skeletal muscle impairment, where damaged contractile tissue was partially replaced by fibrotic and adipose tissue, as demonstrated by the histological and TEM analyses of one-year-old zebrafish. These experiments highlight a dramatic derangement of the muscle tissue with misplaced fiber–fiber contacts as well as fibers presenting a widely variable diameter and fibrosis. Similar features are common in other SG-null animals [13,14,16,17]. The huge number of mononucleated cells, positive to the pan-leukocytic marker L-plastin, confirmed the massive presence of inflammatory cells. The recruitment of cells like macrophages is essential for debris elimination and for the activation of satellite cells supporting muscle regeneration [63]. However, when the degenerative process is massive or at an advanced stage, muscle stem cells are unable to sustain the muscle repair, and the contractile muscle is progressively replaced by fibrotic or adipose tissue [64]. The histological findings were further confirmed at the ultra-structural level. A few of muscle fibers were well-preserved, almost indistinguishable from the wild type, characterized by normal organization of the contractile apparatus, and presented preserved triads, the anatomical basis of skeletal muscle excitation–contraction coupling [65]. In their close proximity, however, the muscle fibers were clearly damaged, with derangement of the sarcomeric array, myofibrillar fragmentations or hypercontraction, and enlargement of the sarcoplasmic reticulum terminal cisternae. Mitochondria in several sections were well-preserved, with no evident difference in comparison to the wild type; on the other hand, mitochondria located in regions of damaged muscle fibers presented a partial detachment of the outer membrane and enlarged intermembrane space. All of these findings suggest a functional mitochondria impairment as a consequence of the calcium overload of damaged fibers, which is in line with observations collected in a variety of animal models of MDs [49,58,66]. 

LGMDR6 is often characterized by a progressive dilated cardiomyopathy that needs constant medical surveillance to prevent possible heart failure [9,67]. In the hamster models of δ-sarcoglycanopathy, both hypertrophic and dilated cardiomyopathies have been described [51,52]. I the *sgcd*^−/−^ zebrafish larvae, no cardiac abnormality was observed. However, the absence of δ-SG from the DAPC in the cardiomyocyte membrane produced signs of cardiomyopathy, evident both macroscopically and microscopically in adulthood. Despite the differences between *D. rerio* and mammals, and although further studies are needed to decipher the type and onset of cardiomyocyte damage, the impairment of both heart and skeletal muscle is proof that the *sgcd*^−/−^ zebrafish phenotype really mirrors the signs, symptoms, and timing of δ-sarcoglycanopathy [2]. 

*Sapje* zebrafish are used for a model of DMD. Dystrophin-deficient larvae rarely survive beyond 14 dpf because they are characterized by severe muscle degeneration with force deficiency [68]. *Sapje* larvae are therefore suitable for performing phenotype-based drug screening campaigns [69,70]. Conversely, the new *sgcd*^−/−^ line, although it can be considered as an excellent model of sarcoglycanopathy, is not directly exploitable at the larval stage for drug screening experiments.

Methods for drug administration in adult zebrafish are available, although they are complex, imprecise, and harmful because they are based on either intraperitoneal, retro-orbital injection, or oral gavage [71,72,73]. Conversely, direct absorption of compounds added in the adult fish water is both imprecise (difficulties in estimating the real amount of adsorbed compound) and may elicit irritation to mucosal surfaces (eyes or gills). Novel methods have been identified for testing compounds in adult animals [74,75], however, the advantage of using zebrafish for drug screening lies precisely in the possibility of performing experiments during the larval stage, when zebrafish can take compounds directly through the skin [18,69,76]. Therefore, to exploit the novel sarcoglycanopathy model, we considered the possibility of anticipating the onset of the myopathic features by growing zebrafish since hatching and up to 5 dpf in 1% methylcellulose dissolved in fish water. The greater than normal effort for swimming in a high-viscosity water, indeed, resulted in the precocious appearance of muscle damage, clearly quantifiable and largely statistically significant by both the birefringence assay and phalloidin staining. Thus, applying such a swimming condition, resembling downhill treadmill running in mice [77], during phenotype-based screening campaigns for new drugs should be straightforward. 

Finally, we evaluated the potential of zebrafish in mimicking sarcoglycanopathy caused by SG missense mutations. For this purpose, we exploited the novel *sgcd^−/−^* line to express the human δ-SG by microinjection in the fertilized oocytes of either the wild type or mutated human mRNAs. It was interesting to observe that at 2–3 dpf, when the injected mRNA is highly translated, the amount of the mutated human protein was lower in comparison to the wild type one. This suggests that the presence of a missense mutation resulted in a folding defective δ-SG, prematurely degraded by ERAD and the ubiquitin-proteasome pathway, like in humans [43,44,45,46]. Indeed, most of the ERAD players have been described in zebrafish and here recognized [78,79,80,81,82]. Furthermore, by blocking the proteasome activity by the inhibitor MG132, we observed an increase in the δ-SG mutant content, in line with data regarding α-SG mutants expressed in mammalian cells [43].

Overall, our findings indicate that sarcoglycanopathy can be effectively modeled in zebrafish. However, unlike what was observed in knockdown experiments and evidence from other lines like the *sapje* mutant, the phenotype of SG-KO lines during the embryonic/larval stages is mild. Nevertheless, these fish develop a progressive muscular dystrophy with severe cardiomyopathy, very well-resembling LGMDR6. Zebrafish also seem useful for developing SG KI lines. Indeed, as ERAD and the ubiquitin-proteasome system are well-conserved in zebrafish, the expression of a missense mutant of δ-SG results in the fast disposal of the protein, which, conversely, is preserved from degradation by inhibiting the proteasome executor. With these positive results, we are confident that the possible future SG-KI lines that we intend to generate will faithfully mimic the forms of sarcoglycanopathy due to the presence of a missense mutation. It is worth mentioning that this kind of mutation accounts for more than 67% of the reported SG genetic defects [5]. 

Taken together, our data pave the way for future studies in which the new KO and future KI lines will be used to gain insight into the first steps of the pathogenic mechanism of sarcoglycanopathies. By way of example and not limited to, we can image calcium dynamics in both skeletal muscle fibers or cardiomyocytes in real-time under basal or stressful conditions (such as swimming in a viscous medium). Similarly, we can monitor mitochondrial function/dysfunction [83]. For this purpose, chemical or genetically encoded probes can be used. In this latter case, the SG-KO lines could be crossed with transgenic zebrafish lines expressing the genetic probes targeted to different organelles and/or expressed in a tissue specific manner [84,85]. This will also be possible thanks to the external development of the organism, the large numbers of individuals at each mating, and the transparency of the embryos and larvae, which allows for direct visualization of the development of tissues and organs such as the muscle and the heart [36]. 

Besides the basic studies, the novel models of sarcoglycanopathy will be of utmost interest in translational research in the field of sarcoglycanopathies, which unfortunately are still orphan diseases. The new SG-KO lines will allow setting up the in vivo screening of novel chemical entities or validated drug, which can result in rescuing or pseudonormalizing the disease phenotype [18]. In other words, we might expect a decrease in muscle damage (measured for example with the birefringence assay) or an increase in the ability to swim under stressful conditions (viscous medium or tunnel-assay), although the evaluation of efficacy will not be limited to these examples. The recovery of the pathologic state can occur through the modulation of the secondary effects of the disease such as inflammation, mitochondrial disfunction, fibrosis, and impaired regeneration. The phenotypic screening is a potent system allowing for the identification of lead compounds, even in the absence of a validated target, or to highlight therapeutics that act on multiple targets, while establishing the safety or toxicity of the tested compounds. The subsequent optimization of the leads can also be assessed through structure–activity relationship (SAR) profiling using the zebrafish models [19].

Despite potential limitations due to the evolutionary distance between *D. rerio* and mammals, we are confident that the new SG-KO lines generated in zebrafish will serve as valuable tools for the scientific community, facilitating significant advancements in the development of effective therapies for sarcoglycanopathies. 

## 4. Material and Methods

### 4.1. Ethics Statement

This study was carried out in accordance with the recommendations in the care and use of laboratory animals of the Italian Parliament L.LGS n26/2014. All procedures on animals were approved by the Italian Ministry of Health n.753/2018-PR.

### 4.2. Zebrafish Husbandry and Maintenance

Zebrafish were maintained in the facility of the University of Padova according to the standard protocols described by [23]. Fish were kept at 28.5 °C in conditioned saline aerated aquaria system under 12 h of light and 12 of dark. For mating, males and females were separated in the late afternoon and were freed the next morning to start courtship, which ended with egg deposition and fecundation. Oocytes were maintained at 28.5 °C in fish water (0.5 mM NaH_2_PO_4_, 0.5 mM NaHPO_4_, 0.2 mg/L methylene blue, 3 mg/L instant ocean pH 7–8) for development.

### 4.3. Generation of Mutated Zebrafish Lines by CRISPR/Cas9 Genome Editing

The sgRNAs targeting either the *sgcd* or *sgcb* gene at the exon2 level were designed by using the Chop-chop software v3 (http://chopchop.cbu.uib.no/ accessed on 10 January 2020). RNA guides with the lowest predicted off target events and the highest matching score for the desired sequences were selected and are reported in Supplementary Materials Table S1. The sgRNAs were produced by using the MEGAScript Kit SP6 (Thermo Fisher Scientific, Ambion, Waltham, MA, USA). Zebrafish WT zygotes were injected with 8 nL of a solution containing 70 ng/μL of sgRNA, 300 ng/µL of Cas9 NLS (Nuclear Localization Signal) protein (New England Biolabs, Ipswich, MA, USA), 1× Phenol red dye, and 1× Danieau injection buffer by using a WPI pneumatic PicoPump PV820 injector. Genomic DNA was extracted from 48 hpf F0 embryos to evaluate the gRNA efficiency in inducing somatic indel mutations. Putative founders were grown to sexual maturity and crossbred with WT. Embryos were raised and genotyped to confirm germline transmission (F1 generation). Heterozygotes were eventually crossbred to obtain the filial generation F2, where the homozygotes (about ¼ of the population) were identified by genotyping and Sanger sequencing.

### 4.4. Genomic DNA Extraction 

The genomic DNA was extracted by using the HotSHOT protocol from the whole embryos at 48 hpf, euthanized by a lethal dose of tricaine (Sigma-Aldrich, St. Louis, MO, USA). For the genotyping of adult zebrafish, the animals were anesthetized with 0.16 mg/mL tricaine solution until gill movement was slowed. A small bioptic fragment from the caudal fin was removed from each fish and the genomic DNA was extracted with the same protocol.

### 4.5. Genotyping of sgcd and sgcb Mutants

Genomic fragments of the targeted regions were amplified by PCR with the 2X DreamTaq Hot Start PCR Master Mix (Thermo Fisher Scientific, K9012) and 0.25 μM of each specific primer designed by the software Primer blast (https://www.ncbi.nlm.nih.gov/tools/primer-blast/index.cgi accessed on 10 January 2020) that are listed in Supplementary Materials Tables S1 and S3. Amplification conditions were as follows: 5 min denaturation at 95 °C, followed by 30 cycles of 30 s at 95 °C, 30 s at 60 °C, 30 s at 72 °C, final extension of 5 min at 72 °C. Mutations in F0 were detected by the heteroduplex mobility shift assay. After DNA amplification, denaturation at 95 °C for 15′ and slow renaturation, the PCR products were run on a 10% 29:1 polyacrylamide gel to observe the presence of heteroduplex bands in comparison to the WT single band. For verification, PCR products from fish harboring indel mutations were sequenced. Poly Peak Parser software (http://yosttools.genetics.utah.edu/PolyPeakParser/ accessed on 10 January 2020) was used for the identification and sequence characterization. Alleles from heterozygous and homozygous fish (F1 and F2) were cloned in pGem-T easy vector (Promega Corporation, Madison, WI, USA) by TA cloning, according to the manufacturer’s instructions, and subsequently used for Sanger sequencing.

### 4.6. Protein Extraction and Western Blot

Pools of 10 embryos/larvae 3 dpf, per lane of SDS-polyacrylamide gel were dissolved in 2X SDS-sample buffer. Two µL of buffer was used per each anesthetized embryo/larva. For the analysis of proteins from the mRNA-injected oocytes, the extraction was performed from 40 larvae by using the GRS FullSample Purification Kit (Grisp, Porto, Portugal), according to the manufacturer’s instructions.

Before loading, the protein lysates were boiled (5 min at 95 °C), then resolved through 10% SDS–PAGE, and blotted to a nitrocellulose membrane (10 mA, 30 min). Membranes were first incubated with the specific primary antibodies listed in Supplementary Materials Table S4, then, after extensive washing, were treated with peroxidase conjugated anti-rabbit or anti-mouse IgG (1:2000) (Sigma Aldrich). WB was developed using ECL turbo (Euroclone, Milan, Italy). The signal from protein bands was detected using an Alliance 9 Mini Chemiluminescence Imaging System (Uvitec, Cambridge, UK). Band intensities were normalized against the total protein loaded (evaluated by Ponceau red staining). 

### 4.7. Body Length Measurement

Body length of the KO and control zebrafish was measured at 3 and 5 dpf and adult animals. Zebrafish were anesthetized by tricaine and digital images were captured using a Leica M80 stereomicroscope fitted with a Leica MC170HD digital camera. Body length was measured from the mouth tip to the tail base along the major body axis in the larvae while both axes were considered in adult fish by using the ImageJ software (version 1.53t).

### 4.8. Phalloidin Staining

Larvae at 5 dpf were fixed in 4% PFA in PBS overnight at 4 °C. Samples were washed in PBS 1X + 0.1% Tween-20 and permeabilized in PBTX 2% (1X PBS + 2% Triton × 100) for 90 min at room temperature. Larvae were bleached with a solution of 3% H_2_0_2_ and 0.35 M of KOH until pigmentation disappeared. After two fast washes in PBTX 0.5%, the larvae were incubated with Phalloidin-iFluor 488 reagent (Abcam, Cambridge, UK) diluted 1:1000 in PBTX 2% overnight at 4 °C. After extensive washing in PBTX 2%, the larvae were embedded in 1.5% low melting agarose on a glass dish for image acquisition using a confocal microscope (Leica TCS SPE, Wetzlar, Germany).

### 4.9. Morphological and Histological Analyses (H&E and Azan–Mallory)

The KO and WT zebrafish were fixed for 48 h in Bouin’s solution at room temperature. Samples were dehydrated through a graded series of ethanol, infiltrated with xylene and included in paraffin (Paraplast plus, Sigma-Aldrich). Samples were serially cut into 10 μm sections on an LKB microtome. After rehydration, the sections were stained for 5 min each in hematoxylin and eosin, dehydrated with ethanol and xylene, and then mounted on a glass coverslip (Lab-tek division, Miles laboratories, Naperville, IL, USA) for microscopic examination.

To evaluate the content of fibrosis, samples were fixed in 4% paraformaldehyde solution for 48 h at 4 °C. To remove calcium salt from the mineralized tissues, the samples were incubated in 0.5 M EDTA solution for 7 days. After the decalcification procedure, the samples were dehydrated in a graded series of ethanol and included in paraffin. Five micrometer-thick sections were cut on a rotary microtome. Sections were then deparaffinized, hydrated, and stained by the Azan–Mallory method to identify collagen fibers. At the end of staining, sections were clarified and mounted in resin for microscopic examination, then the images were acquired by the automated slide scanner (Axioscan 7, Carl Zeiss Microscopy, GmbH, Jena, Germany). 

### 4.10. Heart Dissection

Adult zebrafish were anesthetized and killed according to the standard protocol. Pectoral muscles and fins were removed to open the body cavity; forceps were used to scoop the heart out of the cavity. The extracted hearts were placed in PBS to take pictures.

### 4.11. Birefringence Assay

Anesthetized larvae at 5 dpf placed in 2% methyl cellulose were analyzed between two glass polarizing filters under a Leica M80 stereomicroscope. Larvae were photographed in bright field with a Leica MC170HD digital camera. Pixel intensity in the trunk region was measured by ImageJ software version 1.53t.

### 4.12. Larvae Locomotion Assays

Continuous recording at basal conditions: larvae at 3 dpf were placed in a 96-well plate (1 larva per well, 300 µL of FW medium) in the observation chamber of the DanioVision tracking system (Noldus Information Technology, Wageningen, The Netherlands). Locomotor activity was tracked for about 2 consecutive days and then analyzed by Ethovision 11 software (Noldus Information Technology, NL, Wageningen, The Netherlands). The IR-sensitive camera was set to 25 frames per second. Swimming activity of each larva was calculated as the distance moved during 6 min time windows. A minimal distance movement of 0.2 mm was used. Fish were kept under 12:12 LD cycles (lights on at 08:00, lights off 20:00). 

Light startle test: larvae at 5 dpf were placed in a 24-well plate (1 larva per well, 1 mL of FW medium) in the observation chamber of the DanioVision tracking system (Noldus Information Technology, NL). After 20 min of acclimation in the light, the larvae were subjected to three cycles of 10 min of light followed by 10 min of dark. Swimming activity of each larva was calculated as the total distance moved during 2 min time windows.

### 4.13. Adult Fish Locomotion Assay

From the 7-month-old fish, locomotor activity was video-tracked for 2 days and then analyzed by Ethovision 11 software (Noldus Information Technology, NL). The IR-sensitive camera (Monochrome GigE camera, Basler, Ahrensburg Germany) was set to five frames per second. Locomotor activity of each fish was calculated as the total distance moved during a 6 min time window. A minimal distance movement of 2 mm was used. The IR backlit table (λ > 980 nm) had dimensions of 120 × 120 cm and four fish were placed in an arena of 40 × 40 cm. Fish were kept in 12:12 LD conditions. For the light source, an array of white LED strips (Superlight Technology Co., Ltd., Shenzhen, China) was used, and irradiances were set at 0.17 W/m^2^. Irradiance was measured with a radiometer (DO9721, Probe LP9021 RAD, spectral range 400–950 nm, DeltaOHM, Padova, Italy).

### 4.14. Swimming Tunnel Assay

Fish (N = 19 wild type and N = 24 *sgcd^−/−^*) were not fed for 24 h prior to testing. One fish was randomly selected from the holding aquarium, placed in the swimming chamber, and allowed to recover for 60 min. Water velocity was then increased in steps of 9.6 cm/s at intervals of 10 min until the fish were fatigued and were no longer able to swim (Plaut, 2000). The water temperature during tests was 28 ± 0.5 °C. The chamber size was matched to the species body size. Zebrafish were tested in a channel 0.025 m wide, 0.15 m long, and 0.04 m high. After the test, fish were anesthetized in tricaine and measured for standard body length (BL, measured from the tip of the mouth to the end of the axial musculature). *U_crit_* was calculated using the Brett equation (1964): *U_crit_ = u_i_ +* [*u_s_ × (ti/t)*], where *u_i_* is the highest velocity at which the fish swam during the test, *u_s_* is the velocity step increment (9.6 cm s^−1^), *t_i_* is the time for which the fish swam at the final (fatigue) velocity, and *t* is the time each water velocity was imposed for (10 min).

### 4.15. Electron Microscopy

Euthanized KO and WT larvae at 5 dpf and adults were fixed with Karnovsky fixative (2.5% glutaraldehyde and 2% paraformaldehyde in 0.1 M cacodylate buffer) overnight at 4 °C, washed extensively with 0.1 M cacodylate buffer, post-fixed with osmium tetroxide for 2 h, and embedded in EMbed 812 (Electron Microscopy Sciences, 14,120, Hatfield, PA, USA). Ultrathin sections were stained with uranyl acetate and Pb-citrate and observed with a Philips EM400 operating at 100 kV.

### 4.16. Re-Expression of Human δ-SG cDNA (Wild Type or Mutated) in sgcd^−/−^ Embryos by mRNA Injection of Fertilized Oocytes

For the preparation of the δ-SG human sequences, the pCDNA3.1 DYK (flag tag) vector expressing the full length human δ-SG was purchased from Genscript Biotech B.V., Leiden, Netherlands. The coding sequence of δ-SG was transferred from pcDNA3.1 DYK to the pCS2+ vector by PCR amplification with primers harboring appropriate restriction enzyme site sequences. All mutagenesis reactions were performed with the GeneArt Site Directed Mutagenesis Kit (Thermo Fisher Scientific) according to the manufacturer’s instructions. All constructs were verified by sequencing. Mutagenesis to generate the human E262K-δ-SG was performed with the following mutagenic primers: Fwd 5′-AGGCAGAAGGTCTTCaAGATCTGCGTCTGCG-3′; Rev 5′-CGCAGACGCAGATCTtGAAGACCTTCTGCCT-3′. All constructs were verified by Sanger sequencing. 

For the preparation of mRNA, the pCS2+ vectors expressing the full-length sequence of either the WT or mutated δ-SG were linearized with NotI, and in vitro transcribed with the Sp6 mMESSAGE mMACHINE Kit (Thermo Fisher Scientific), according to the manufacturer’s instructions. The resulting capped mRNAs were purified by LiCl precipitation and re-suspended in nuclease free water. The quality of the mRNAs was evaluated by running an aliquot on a denaturing 8 M urea polyacrylamide gel. The mRNA concentration was determined by UV spectroscopy with a Nanodrop spectrophotometer (Thermo Fisher Scientific). Different mRNA concentrations (50, 100, 150 ng/μL) were injected into the *sgcd*^−/−^ fertilized oocytes using 1X Danieu injection buffer containing 1X phenol red dye. The injection was performed using a WPI pneumatic PicoPump PV820 injector.

For MG132 treatment, eight hours post mRNA injection, 10 µM MG132 (Sigma-Aldrich) or its vehicle DMSO 1‰ was added to the fish water of the developing embryos. After 40 h of treatment, the embryos were lysed in 2X SDS-sample buffer and proteins were analyzed by SDS-PAGE and WB, as above described.

### 4.17. Statistical Analysis

Data were expressed as the mean ± SD. Statistical differences among groups (control vs. mutated) were determined using Pris, Version 10.0.2 GraphPad software (San Diego, CA, USA) by the non-parametric Mann–Whitney test. If more than two groups were compared, statistical differences were determined by the one-way ANOVA test, followed by Tukey’s multiple comparisons test. A level of confidence of *p* < 0.05 was used for statistical significance.

## Data Availability

The data presented in this study are available in the article and in the Supplementary Materials.

## Supplementary Materials

The following supporting information can be downloaded at: https://www.mdpi.com/article/10.3390/ijms241612707/s1.

## References

[1] Straub V., Murphy A., Udd B., LGMD Workshop Study Group (2018). 229th ENMC international workshop: Limb girdle muscular dystrophies—Nomenclature and reformed classification Naarden, The Netherlands, 17–19 March 2017. Neuromuscul. Disord..

[2] Vainzof M., Souza L.S., Gurgel-Giannetti J., Zatz M. (2021). Sarcoglycanopathies: An update. Neuromuscul. Disord..

[3] Ozawa E. (2010). Our trails and trials in the subsarcolemmal cytoskeleton network and muscular dystrophy researches in the dystrophin era. Proc. Jpn. Acad. Ser. B Phys. Biol. Sci..

[4] Tarakci H., Berger J. (2016). The sarcoglycan complex in skeletal muscle. Front. Biosci. (Landmark Ed.).

[5] Carotti M., Fecchio C., Sandona D. (2017). Emerging therapeutic strategies for sarcoglycanopathy. Expert Opin. Orphan D.

[6] Angelini C., Fanin M. (2016). Pathogenesis, clinical features and diagnosis of sarcoglycanopathies. Expert Opin Orphan D.

[7] Alonso-Perez J., Gonzalez-Quereda L., Bello L., Guglieri M., Straub V., Gallano P., Semplicini C., Pegoraro E., Zangaro V., Nascimento A. (2020). New genotype-phenotype correlations in a large European cohort of patients with sarcoglycanopathy. Brain.

[8] Kirschner J., Lochmuller H. (2011). Sarcoglycanopathies. Handb. Clin. Neurol.

[9] Politano L., Nigro V., Passamano L., Petretta V., Comi L.I., Papparella S., Nigro G., Rambaldi P.F., Raia P., Pini A. (2001). Evaluation of cardiac and respiratory involvement in sarcoglycanopathies. Neuromuscul. Disord..

[10] Fanin M., Melacini P., Boito C., Pegoraro E., Angelini C. (2003). LGMD2E patients risk developing dilated cardiomyopathy. Neuromuscul. Disord..

[11] Ng R., Banks G.B., Hall J.K., Muir L.A., Ramos J.N., Wicki J., Odom G.L., Konieczny P., Seto J., Chamberlain J.R. (2012). Animal Models of Muscular Dystrophy. Prog. Mol. Biol. Transl..

[12] Gaina G., Popa Gruianu A. (2021). Muscular dystrophy: Experimental animal models and therapeutic approaches (Review). Exp. Ther. Med..

[13] Durbeej M., Cohn R.D., Hrstka R.F., Moore S.A., Allamand V., Davidson B.L., Williamson R.A., Campbell K.P. (2000). Disruption of the beta-sarcoglycan gene reveals pathogenetic complexity of limb-girdle muscular dystrophy type 2E. Mol. Cell.

[14] Hack A.A., Cordier L., Shoturma D.I., Lam M.Y., Sweeney H.L., McNally E.M. (1999). Muscle degeneration without mechanical injury in sarcoglycan deficiency. Proc. Natl. Acad. Sci. USA.

[15] Coral-Vazquez R., Cohn R.D., Moore S.A., Hill J.A., Weiss R.M., Davisson R.L., Straub V., Barresi R., Bansal D., Hrstka R.F. (1999). Disruption of the sarcoglycan-sarcospan complex in vascular smooth muscle: A novel mechanism for cardiomyopathy and muscular dystrophy. Cell.

[16] Nigro V., Okazaki Y., Belsito A., Piluso G., Matsuda Y., Politano L., Nigro G., Ventura C., Abbondanza C., Molinari A.M. (1997). Identification of the Syrian hamster cardiomyopathy gene. Hum. Mol. Genet..

[17] Duclos F., Straub V., Moore S.A., Venzke D.P., Hrstka R.F., Crosbie R.H., Durbeej M., Lebakken C.S., Ettinger A.J., van der Meulen J. (1998). Progressive muscular dystrophy in alpha-sarcoglycan-deficient mice. J. Cell Biol..

[18] Patton E.E., Zon L.I., Langenau D.M. (2021). Zebrafish disease models in drug discovery: From preclinical modelling to clinical trials. Nat. Rev. Drug Discov..

[19] Rosa J.G.S., Lima C., Lopes-Ferreira M. (2022). Zebrafish Larvae Behavior Models as a Tool for Drug Screenings and Pre-Clinical Trials: A Review. Int. J. Mol. Sci..

[20] Steffen L.S., Guyon J.R., Vogel E.D., Beltre R., Pusack T.J., Zhou Y., Zon L.I., Kunkel L.M. (2007). Zebrafish orthologs of human muscular dystrophy genes. BMC Genom..

[21] Medishetti R., Balamurugan K., Yadavalli K., Rani R., Sevilimedu A., Challa A.K., Parsa K., Chatti K. (2022). CRISPR-Cas9-induced gene knockout in zebrafish. STAR Protoc..

[22] Hermsen S.A., van den Brandhof E.J., van der Ven L.T., Piersma A.H. (2011). Relative embryotoxicity of two classes of chemicals in a modified zebrafish embryotoxicity test and comparison with their in vivo potencies. Toxicol. Vitr..

[23] Kimmel C.B., Ballard W.W., Kimmel S.R., Ullmann B., Schilling T.F. (1995). Stages of embryonic development of the zebrafish. Dev. Dyn..

[24] Guyon J.R., Mosley A.N., Jun S.J., Montanaro F., Steffen L.S., Zhou Y., Nigro V., Zon L.I., Kunkel L.M. (2005). Delta-sarcoglycan is required for early zebrafish muscle organization. Exp. Cell Res..

[25] Cheng L., Guo X.F., Yang X.Y., Chong M., Cheng J., Li G., Gui Y.H., Lu D.R. (2006). Delta-sarcoglycan is necessary for early heart and muscle development in zebrafish. Biochem. Biophys. Res. Commun..

[26] Best C., Kurrasch D.M., Vijayan M.M. (2017). Maternal cortisol stimulates neurogenesis and affects larval behaviour in zebrafish. Sci. Rep..

[27] Di Rosa V., Frigato E., Lopez-Olmeda J.F., Sanchez-Vazquez F.J., Bertolucci C. (2015). The Light Wavelength Affects the Ontogeny of Clock Gene Expression and Activity Rhythms in Zebrafish Larvae. PLoS ONE.

[28] Han E., Ho Oh K., Park S., Chan Rah Y., Park H.C., Koun S., Choi J. (2020). Analysis of behavioral changes in zebrafish (*Danio rerio*) larvae caused by aminoglycoside-induced damage to the lateral line and muscles. Neurotoxicology.

[29] Zarantoniello M., Randazzo B., Gioacchini G., Truzzi C., Giorgini E., Riolo P., Gioia G., Bertolucci C., Osimani A., Cardinaletti G. (2020). Zebrafish (*Danio rerio*) physiological and behavioural responses to insect-based diets: A multidisciplinary approach. Sci. Rep..

[30] Morbiato E., Bilel S., Tirri M., Arfe R., Fantinati A., Savchuk S., Appolonova S., Frisoni P., Tagliaro F., Neri M. (2020). Potential of the zebrafish model for the forensic toxicology screening of NPS: A comparative study of the effects of APINAC and methiopropamine on the behavior of zebrafish larvae and mice. Neurotoxicology.

[31] Lucon-Xiccato T., Tomain M., D’Aniello S., Bertolucci C. (2023). bdnf loss affects activity, sociability, and anxiety-like behaviour in zebrafish. Behav. Brain Res..

[32] MacPhail R.C., Brooks J., Hunter D.L., Padnos B., Irons T.D., Padilla S. (2009). Locomotion in larval zebrafish: Influence of time of day, lighting and ethanol. Neurotoxicology.

[33] Smith L.L., Beggs A.H., Gupta V.A. (2013). Analysis of skeletal muscle defects in larval zebrafish by birefringence and touch-evoke escape response assays. J. Vis. Exp..

[34] Blain A.M., Straub V.W. (2011). δ-Sarcoglycan-deficient muscular dystrophy: From discovery to therapeutic approaches. Skelet. Muscle.

[35] Xie Z., Hou Y., Yu M., Liu Y., Fan Y., Zhang W., Wang Z., Xiong H., Yuan Y. (2019). Clinical and genetic spectrum of sarcoglycanopathies in a large cohort of Chinese patients. Orphanet J. Rare Dis..

[36] Tesoriero C., Greco F., Cannone E., Ghirotto F., Facchinello N., Schiavone M., Vettori A. (2023). Modeling Human Muscular Dystrophies in Zebrafish: Mutant Lines, Transgenic Fluorescent Biosensors, and Phenotyping Assays. Int. J. Mol. Sci..

[37] Lucon-Xiccato T., Bella L., Mainardi E., Baraldi M., Bottarelli M., Sandona D., Bertolucci C. (2021). An Automated Low-Cost Swim Tunnel for Measuring Swimming Performance in Fish. Zebrafish.

[38] Wakamatsu Y., Ogino K., Hirata H. (2019). Swimming capability of zebrafish is governed by water temperature, caudal fin length and genetic background. Sci. Rep..

[39] Hu N., Sedmera D., Yost H.J., Clark E.B. (2000). Structure and function of the developing zebrafish heart. Anat. Rec..

[40] Hu N., Yost H.J., Clark E.B. (2001). Cardiac morphology and blood pressure in the adult zebrafish. Anat. Rec..

[41] Muntoni F. (2003). Cardiomyopathy in muscular dystrophies. Curr. Opin. Neurol..

[42] Ruparelia A.A., Oorschot V., Vaz R., Ramm G., Bryson-Richardson R.J. (2014). Zebrafish models of BAG3 myofibrillar myopathy suggest a toxic gain of function leading to BAG3 insufficiency. Acta Neuropathol..

[43] Gastaldello S., D’Angelo S., Franzoso S., Fanin M., Angelini C., Betto R., Sandona D. (2008). Inhibition of proteasome activity promotes the correct localization of disease-causing alpha-sarcoglycan mutants in HEK-293 cells constitutively expressing beta-, gamma-, and delta-sarcoglycan. Am. J. Pathol..

[44] Bartoli M., Gicquel E., Barrault L., Soheili T., Malissen M., Malissen B., Vincent-Lacaze N., Perez N., Udd B., Danos O. (2008). Mannosidase I inhibition rescues the human alpha-sarcoglycan R77C recurrent mutation. Hum. Mol. Genet..

[45] Bianchini E., Fanin M., Mamchaoui K., Betto R., Sandona D. (2014). Unveiling the degradative route of the V247M alpha-sarcoglycan mutant responsible for LGMD-2D. Hum. Mol. Genet..

[46] Soheili T., Gicquel E., Poupiot J., N’Guyen L., Le Roy F., Bartoli M., Richard I. (2012). Rescue of sarcoglycan mutations by inhibition of endoplasmic reticulum quality control is associated with minimal structural modifications. Hum. Mutat..

[47] Winder S.J., Lipscomb L., Angela Parkin C., Juusola M. (2011). The proteasomal inhibitor MG132 prevents muscular dystrophy in zebrafish. PLoS Curr..

[48] Mareedu S., Million E.D., Duan D., Babu G.J. (2021). Abnormal Calcium Handling in Duchenne Muscular Dystrophy: Mechanisms and Potential Therapies. Front. Physiol..

[49] Zulian A., Schiavone M., Giorgio V., Bernardi P. (2016). Forty years later: Mitochondria as therapeutic targets in muscle diseases. Pharmacol. Res..

[50] Vainzof M., Ayub-Guerrieri D., Onofre P.C., Martins P.C., Lopes V.F., Zilberztajn D., Maia L.S., Sell K., Yamamoto L.U. (2008). Animal models for genetic neuromuscular diseases. J. Mol. Neurosci..

[51] Sakamoto A., Ono K., Abe M., Jasmin G., Eki T., Murakami Y., Masaki T., Toyo-oka T., Hanaoka F. (1997). Both hypertrophic and dilated cardiomyopathies are caused by mutation of the same gene, delta-sarcoglycan, in hamster: An animal model of disrupted dystrophin-associated glycoprotein complex. Proc. Natl. Acad. Sci. USA.

[52] Mitsuhashi S., Saito N., Watano K., Igarashi K., Tagami S., Shima H., Kikuchi K. (2003). Defect of delta-sarcoglycan gene is responsible for development of dilated cardiomyopathy of a novel hamster strain, J2N-k: Calcineurin/PP2B activity in the heart of J2N-k hamster. J. Biochem..

[53] Henriques S.F., Patissier C., Bourg N., Fecchio C., Sandona D., Marsolier J., Richard I. (2018). Different outcome of sarcoglycan missense mutation between human and mouse. PLoS ONE.

[54] Kobuke K., Piccolo F., Garringer K.W., Moore S.A., Sweezer E., Yang B., Campbell K.P. (2008). A common disease-associated missense mutation in alpha-sarcoglycan fails to cause muscular dystrophy in mice. Hum. Mol. Genet..

[55] Howe K., Clark M.D., Torroja C.F., Torrance J., Berthelot C., Muffato M., Collins J.E., Humphray S., McLaren K., Matthews L. (2013). The zebrafish reference genome sequence and its relationship to the human genome. Nature.

[56] Li Y., Jia Z., Zhang S., He X. (2021). Progress in Gene-Editing Technology of Zebrafish. Biomolecules.

[57] Burgess H.A., Granato M. (2007). Modulation of locomotor activity in larval zebrafish during light adaptation. J. Exp. Biol..

[58] Schiavone M., Zulian A., Menazza S., Petronilli V., Argenton F., Merlini L., Sabatelli P., Bernardi P. (2017). Alisporivir rescues defective mitochondrial respiration in Duchenne muscular dystrophy. Pharmacol. Res..

[59] El-Brolosy M.A., Kontarakis Z., Rossi A., Kuenne C., Gunther S., Fukuda N., Kikhi K., Boezio G.L.M., Takacs C.M., Lai S.L. (2019). Genetic compensation triggered by mutant mRNA degradation. Nature.

[60] Joris M., Schloesser M., Baurain D., Hanikenne M., Muller M., Motte P. (2017). Number of inadvertent RNA targets for morpholino knockdown in *Danio rerio* is largely underestimated: Evidence from the study of Ser/Arg-rich splicing factors. Nucleic Acids Res..

[61] Kok F.O., Shin M., Ni C.W., Gupta A., Grosse A.S., van Impel A., Kirchmaier B.C., Peterson-Maduro J., Kourkoulis G., Male I. (2015). Reverse genetic screening reveals poor correlation between morpholino-induced and mutant phenotypes in zebrafish. Dev. Cell.

[62] Krylov V.V., Izvekov E.I., Pavlova V.V., Pankova N.A., Osipova E.A. (2021). Circadian rhythms in zebrafish (*Danio rerio*) behaviour and the sources of their variability. Biol. Rev. Camb. Philos. Soc..

[63] Wang X., Zhou L. (2022). The Many Roles of Macrophages in Skeletal Muscle Injury and Repair. Front. Cell Dev. Biol..

[64] Smith L.R., Barton E.R. (2018). Regulation of fibrosis in muscular dystrophy. Matrix Biol..

[65] Al-Qusairi L., Laporte J. (2011). T-tubule biogenesis and triad formation in skeletal muscle and implication in human diseases. Skelet. Muscle.

[66] Bellissimo C.A., Garibotti M.C., Perry C.G.R. (2022). Mitochondrial stress responses in Duchenne muscular dystrophy: Metabolic dysfunction or adaptive reprogramming?. Am. J. Physiol. Cell Physiol..

[67] Schade van Westrum S.M., Dekker L.R., de Voogt W.G., Wilde A.A., Ginjaar I.B., de Visser M., van der Kooi A.J. (2014). Cardiac involvement in Dutch patients with sarcoglycanopathy: A cross-sectional cohort and follow-up study. Muscle Nerve.

[68] Widrick J.J., Alexander M.S., Sanchez B., Gibbs D.E., Kawahara G., Beggs A.H., Kunkel L.M. (2016). Muscle dysfunction in a zebrafish model of Duchenne muscular dystrophy. Physiol. Genom..

[69] Farr G.H., Morris M., Gomez A., Pham T., Kilroy E., Parker E.U., Said S., Henry C., Maves L. (2020). A novel chemical-combination screen in zebrafish identifies epigenetic small molecule candidates for the treatment of Duchenne muscular dystrophy. Skelet. Muscle.

[70] Kawahara G., Karpf J.A., Myers J.A., Alexander M.S., Guyon J.R., Kunkel L.M. (2011). Drug screening in a zebrafish model of Duchenne muscular dystrophy. Proc. Natl. Acad. Sci. USA.

[71] Dang M., Henderson R.E., Garraway L.A., Zon L.I. (2016). Long-term drug administration in the adult zebrafish using oral gavage for cancer preclinical studies. Dis. Model Mech..

[72] Kinkel M.D., Eames S.C., Philipson L.H., Prince V.E. (2010). Intraperitoneal injection into adult zebrafish. J. Vis. Exp..

[73] Pugach E.K., Li P., White R., Zon L. (2009). Retro-orbital injection in adult zebrafish. J. Vis. Exp..

[74] Chang C.T., Doerr K.M., Whipps C.M. (2017). Antibiotic treatment of zebrafish mycobacteriosis: Tolerance and efficacy of treatments with tigecycline and clarithromycin. J. Fish Dis..

[75] Lu Y., Patton E.E. (2022). Long-term non-invasive drug treatments in adult zebrafish that lead to melanoma drug resistance. Dis. Model Mech..

[76] Kawahara G., Kunkel L.M. (2013). Zebrafish based small molecule screens for novel DMD drugs. Drug Discov. Today Technol..

[77] Hyzewicz J., Ruegg U.T., Takeda S. (2015). Comparison of Experimental Protocols of Physical Exercise for mdx Mice and Duchenne Muscular Dystrophy Patients. J. Neuromuscul. Dis..

[78] Barbieri A., Carra S., De Blasio P., Cotelli F., Biunno I. (2018). Sel1l knockdown negatively influences zebrafish embryos endothelium. J. Cell Physiol..

[79] Chen Z., Ballar P., Fu Y., Luo J., Du S., Fang S. (2014). The E3 ubiquitin ligase gp78 protects against ER stress in zebrafish liver. J. Genet. Genom..

[80] Imamura S., Yabu T., Yamashita M. (2012). Protective role of cell division cycle 48 (CDC48) protein against neurodegeneration via ubiquitin-proteasome system dysfunction during zebrafish development. J. Biol. Chem..

[81] Imai F., Yoshizawa A., Fujimori-Tonou N., Kawakami K., Masai I. (2010). The ubiquitin proteasome system is required for cell proliferation of the lens epithelium and for differentiation of lens fiber cells in zebrafish. Development.

[82] Wang Y., Fu X., Gaiser S., Kottgen M., Kramer-Zucker A., Walz G., Wegierski T. (2007). OS-9 regulates the transit and polyubiquitination of TRPV4 in the endoplasmic reticulum. J. Biol. Chem..

[83] Stocco A., Smolina N., Sabatelli P., Šileikytė J., Artusi E., Mouly V., Cohen M., Forte M., Schiavone M., Bernardi P. (2021). Treatment with a triazole inhibitor of the mitochondrial permeability transition pore fully corrects the pathology of sapje zebrafish lacking dystrophin. Pharmacol. Res..

[84] Kettunen P., Islam M. (2020). Calcium Imaging in the Zebrafish. Calcium Signaling.

[85] Salgado-Almario J., Vicente M., Molina Y., Martinez-Sielva A., Vincent P., Domingo B., Llopis J. (2022). Simultaneous imaging of calcium and contraction in the beating heart of zebrafish larvae. Theranostics.

