# Supplementary material for: Modeling Sarcoglycanopathy in Danio rerio
Source: Int J Mol Sci. 2023 Aug 11;24(16):12707. doi: 10.3390/ijms241612707 (PMC10454440; doi:10.3390/ijms241612707)
Supplement: Supplementary file 1 [file ijms-24-12707-s001.zip › ijms-2533093-Supplementary_material.pdf]

Supplementary material

Modelling sarcoglycanopathy in *Danio rerio*

Francesco Dalla Barba, Michela Soardi, Leila Mouhib, Giovanni Risato, Eylem Emek Akyurek, Tyrone Lucon-Xiccato, Martina Scano, Alberto Benetollo, Roberta Sacchetto, Isabelle Richard, Francesco Argenton, Cristiano Bertolucci, Marcello Carotti, Dorianna Sandonà

Table S1

Table S1. sgRNAs and primers for genome editing and screening

**NAME**	**sequence**
**δ-SG$_{EX2}$KOGUIDE**	GAGTGGGGATCTACGGCTGG
**β-SG$_{EX2}$KOGUIDE**	GAGGAGAGACTCCACAAGAC
**δ-SG$_{EX2}$KO_FW**	5’-ACAGTGCCCTCATAGGAACAAT-3’
**δ-SG$_{EX2}$KO_RV**	5’-CCTCTAAAGGCGGTTCAAAGAA-3’
**β-SG$_{EX2}$KO-FW**	5’-CAAATGTCCACAGGAAATCTCA-3’
**β-SG$_{EX2}$KO-RV**	5’-AATGAGAGCAAGCAGAAAAAGC-3’
**δ-SG$_{EX2}$KO_SCREENWT_FW**	5’-TGTTGTGTTGCATCTTTGCATTTTGG-3’
**δ-SG$_{EX2}$KO_SCREENRV**	5’-GCAACGTTTCCGCCAGCC-3’
**δ-SG$_{EX2}$KO_SCREENMUT_FW**	5’-GGCAACGTTTCCGCCGTAG-3’
**β-SG$_{EX2}$KO_SCREENWT_FW**	5’-GGAGAGACTCCACAAGACAGGA-3’
**β-SG$_{EX2}$KO_SCREENRV**	5’-CTGCATCTCTAGGAGTTTGACTGT-3’
**β-SG$_{EX2}$KO_SCREENMUT_FW**	5’-GAGGAGAGACTCCACAGACTCC-3’

Analysis of off-targets in *sgcd*$^{-/-}$ zebrafish line

We examined whether the CRISPR/Cas9 generated off-target mutations in the zebrafish genome.
Putative off-target sites were identified by a base-by-base scan of the entire zebrafish genome allowing for gapped and ungapped alignments with up to 4 mismatches in the sgRNA target sequence (Table S2) using the Chop-chop v3 software (http://chopchop.cbu.uib.no/ accessed on 10 January 2020). Five genes were identified as a possible unwanted target of CRISPR/Cas9 based on the sequence of δ-SG$_{ex2}$KO guide (Table S2). Genes involved are: *smyd3* (SET and MYND domain containing 3), *sgcg* (sarcoglycan, gamma), *tmc1* (transmembrane channel-like 1), *asap1a* (ArfGAP with SH3 domain, ankyrin repeat and PH domain 1a), *ppp1r13l* (protein phosphatase 1, regulatory subunit 13 like). Regions identified as a possible off-targets in each individual gene were then amplified by PCR and sequenced. Primers used for the PCR are reported in Table S3. The results of the Sanger sequencing showed that none of these sequences was modified by the action of CRISPR/Cas9.

Table S2: Sequences predicted to be recognised by δ-SG$_{ex2}$KO guide to induce off-targets.
For each sequences the PAM region, number of mismatch (# MM), genes and locus involved are indicated.

Sequence	PAM	#MM	Gene	Locus
CGGTGGGGATCCTCGGCTGG	AGG	4	SMYD3	17:+12018737
GA-TCGGCATCTACGGCTGG	AGG	3	SGCG	15:+9332196
GCCTGGAGAACTACGGCTGG	TGG	4	TMC1	5:-25661131
GAGTGGA-ATCTGCGGCTGG	AGG	3	ASAP1A	2:-32280016
AAGTGAGC-TCTACGGCTGG	CAG	4	PPP1R13L	18:-35886279

Table S3: primer sequences used to amplify regions predicted as off targets.

PRIMER	SEQUENCE
***smyd3*_FW**	5’-GTCAATGTCAAACCAGCCCG-3’
***smyd3*_RV**	5’-TACAGCTCCTCCTGGTCACT-3'
***sgcg*_FW**	5’-CTTGTTTGGCAGATGGTGCGT-3’
***sgcg* _RV**	5’-CTTGTTTGGCAGATGGTGCGT-3’
***tmc1*_FW**	5’-GTGGAGGAGCAAGAGAGCAG-3’
***tmc1*_RV**	5’-GCATCCATGAGGGCGATGAT-3’
***asap1a*_FW**	5’-ATCAAAACGGGGAGACAGCAT-3’
***asap1a*_RV**	5’-TTGTACTTCTCATGCAGCGT-3’
***ppp1r13l*_FW**	5’-GCATCAGGGAAGAACCAGCAG-3’
ppp1r13l_RV	5’-AACGCCACAGCATTTTCTGATT-3’

Figure S1

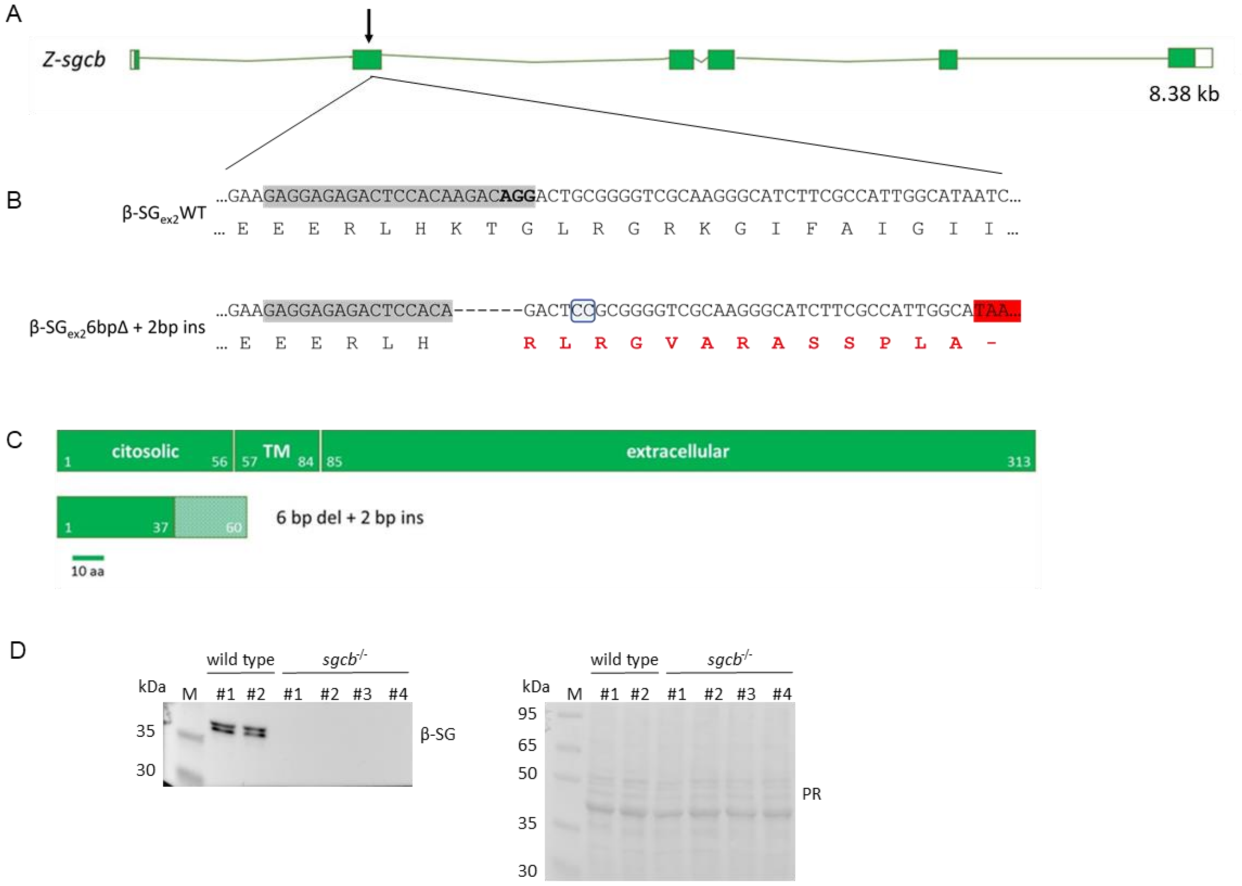

Figure S1. β-KOex2 zebrafish, mutation introduced in the z-sgcb gene and consequences at the protein level. *(A) Genomic organization of the wild type z-sgcb gene: boxes, exons; lines, introns. The arrow points the site in exon2 targeted by the Cas9. (B) Nucleotide and amino acid sequences of wild type and the mutant (6-bp deletion +2-bp insertion) as revealed by DNA sequencing analysis of the sgcb CRISPR target site. Each deleted nucleotide is represented by a dash, while the 2 nucleotides inserted are boxed, CRISPR target site is highlighted in grey with the PAM sequence in bold. The amino acid sequences of the wild type (black amino acid) and of the mutant is reported under the nucleotide sequence. The consequence of the mutations is a frame shift (red amino acids) with the appearance of a premature stop codon. (C) Scheme of the primary sequence of the wild type β-SG protein and of the predicted truncated form deriving from the expression of the mutated version of the sgcb gene, with the different topological domains. (D) Western blot analysis showing the absence of the β-SG protein in the lysates coming from embryos of the β-KOex2 zebrafish line (sgcb$^{-/-}$). Five zebrafish embryos at 3 dpf of the different samples were lysed and loaded into SDS PAGE. After blotting, proteins were probed with primary antibody recognizing the β-SG protein. Lysates from wild type zebrafish embryos were used as positive controls.*

Figure S2

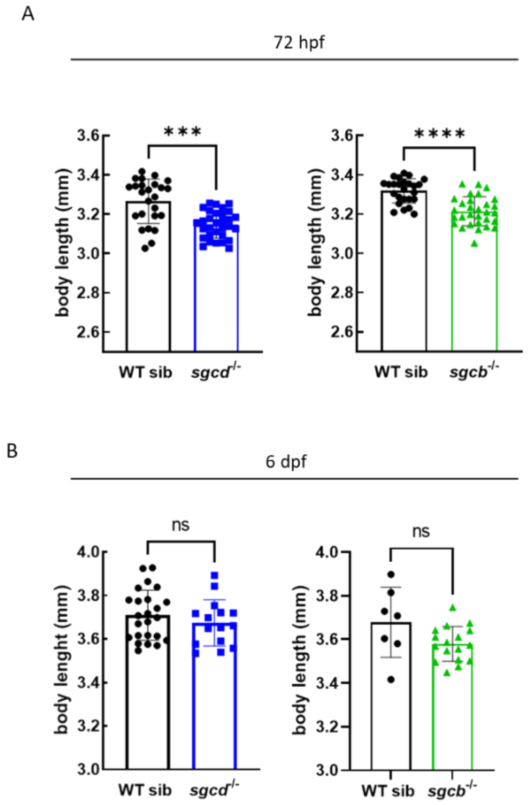

C

	normal 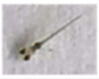	curved spine 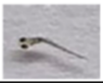
wild type sib	93.5%	6.5%
sgcd $^{-/-}$	86.8%	13.2%
wild type sib	98.3%	1.7%
sgcb $^{-/-}$	83.3%	16.7%

7 dpf

Figure S2. Length and morphology of sgcd $^{-/-}$ ***and sgcb*** $^{-/-}$ ***larvae.*** *(A) Body length of the sgcd* $^{-/-}$ *and sgcb* $^{-/-}$ *larvae, at 72 hpf, is slightly shorter than the wild type one. (B) Conversely, no statistically significant difference was observed in comparison to the wild type at 6 dpf. Statistical analysis was performed by Mann-Whitney test; ***, P ≤ 0.001; ****, P ≤ 0.0001. (C) Table reporting the percentage of larvae of sgcd* $^{-/-}$*, sgcb* $^{-/-}$ *and wild type siblings at 7 dpf with normal or altered phenotype.*

Figure S3

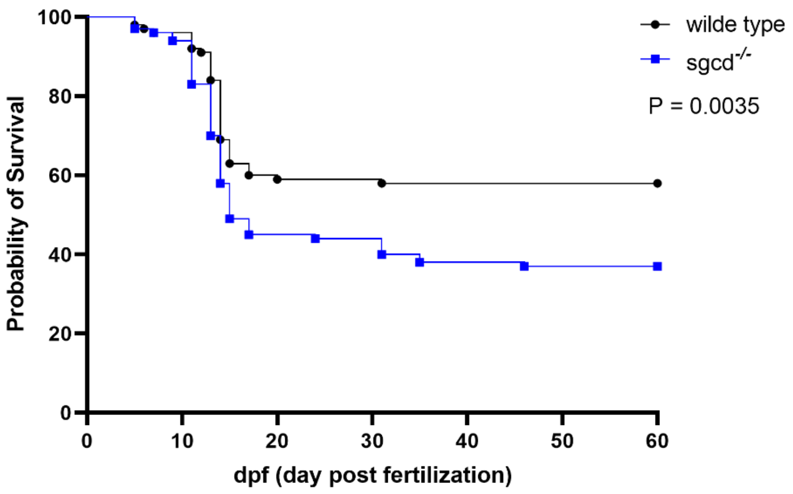

Figure S3 Kaplan-Mayer analysis of the sgcd$^{-/-}$ and wild type zebrafish. *It is clear that during the first 7-10 days post fertilization, mutants and wild type zebrafish behaved similarly. On the other hand, when fish started to feed autonomously, it is possible to observe a divergence in the curves, with mutants showing a reduced survival rate (about 40%) that is statistically significant different in comparison to wild type fish (about 60%).*

Figure S4

A

δ-SG$_{ATG}$MO-2	3'-CAGTCCTACTACTGGGTCCTTGTCA-5'
δ-SG mRNAzebrafish	5'- AUGGUCAGGAUGAUGACCCAGGAACAGUGCCA – 3'

B

Std-MO: 5'-CCTCTTACCTCAGTTACAATTTATA-3'

δ-SG$_{ATG}$MO: 5'-ACTGTTCCTGGGTCATCATCCTGAC-3'

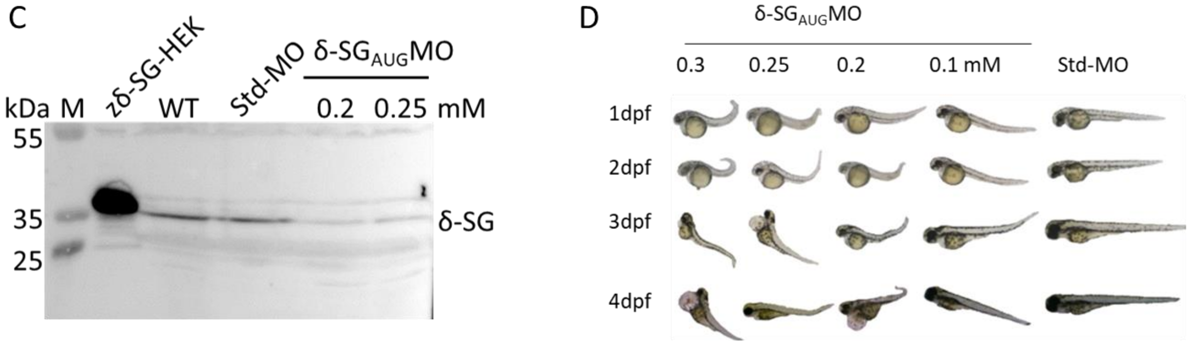

E

oligo	total	death	surviving at 4dpf	% death	% alive	% without pheno-type (normal/ alive)	% with phenotype (altered/alive)
StdMO CTRL	272	49	223	18.01%	81.99%	100%	0.00%
δ-SG$_{ATG}$MO 0.1 mM	367	138	229	37.60%	62.40%	90.83%	9.17%
δ-SG$_{ATG}$MO 0.2 mM	226	115	111	50.88%	49.12%	72.97%	27.03%
δ-SG$_{ATG}$MO 0.25mM	389	207	182	53.21%	46.79%	63.74%	36.26%
δ-SG$_{ATG}$MO 0.3 mM	225	131	94	58.22%	41.78%	56.38%	43.62%

Figure S4: sgcd knock down by morpholino oligo

(A) Region of the zebrafish sgcd mRNA (δ-SG-mRNA), encompassing the AUG codon, targeted by the δ-SG morpholino oligo (δ-SG$_{ATG}$-MO). (B) sequences of the standard oligonucleotide (StdMO) used as negative control and of the δ-SG$_{ATG}$-MO. These MOs were microinjected in 1 cell-stage fertilized oocytes. (C) Western blot of proteins extracts from 3 dpf larvae of either un-injected wild type or injected with std-MO or δ-SG$_{AUG}$MO zebrafish. As positive control, proteins from HEK293 cells transiently transfected with a vector expressing the zebrafish δ-SG sequence (zδ-SG-HEK293) were loaded in the gel. Western blot analysis was performed using rabbit polyclonal anti-δ-SG antibodies. (D) representative images of the altered morphology of zebrafish at the indicated time points, injected with δ-SG$_{AUG}$MO, at the reported concentrations; representative images of zebrafish injected with the Std-MO 0.25 mM are reported for comparison. Embryos and larvae in which δ-SG protein was knocked down showed a curved or bent phenotype, suggesting muscle impairment. (E) table reporting a quantitative analysis of the effects of δ-SG knock down by increasing concentration of MO.

Figure S5

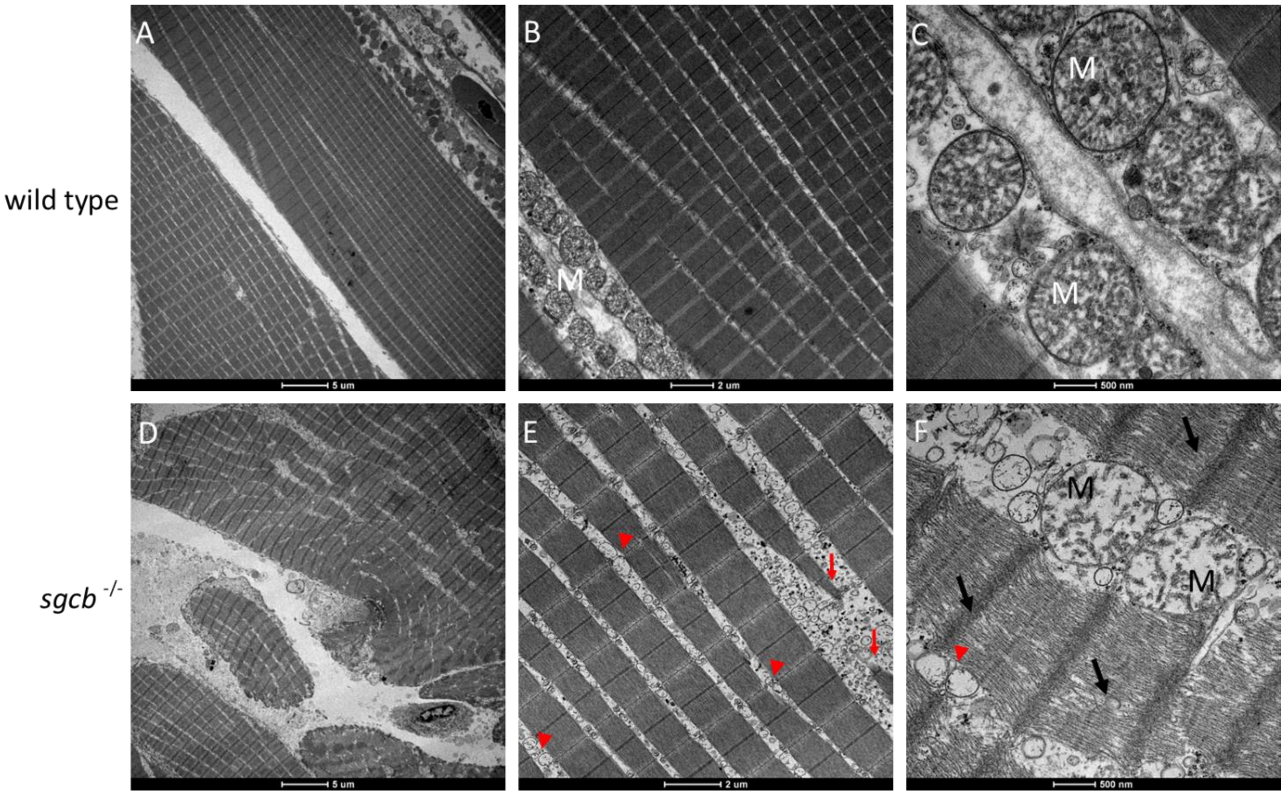

Figure S5: Transmission electron microscopy analysis of skeletal muscle of WT and sgcb$^{-/-}$ adult zebrafish lines.

WT (panel A-C) displayed well-organized myofibrils with well-preserved mitochondria (M). On the contrary sgcb$^{-/-}$ (panel D-F) presented less compacted, fragmented (red arrows) or waived myofibrils (black arrows) and dilated terminal cisternae (red arrowheads). Mitochondria in regions of damaged fibers appeared dilated with hypodense matrix and reduced cristae organization. Scale bar: um, µm.

Figure S6

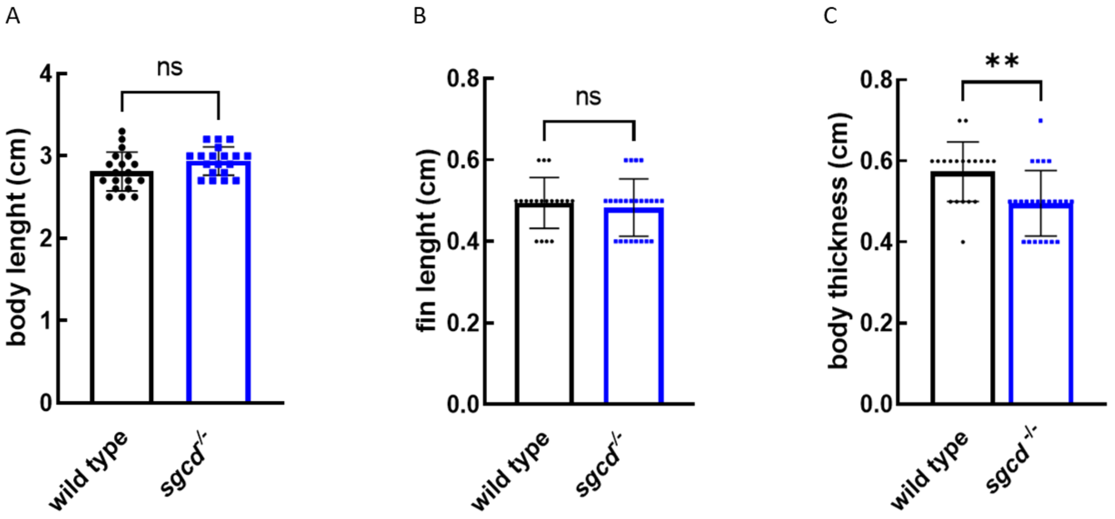

Figure S6: morphometric analysis of 1-year-old wild type and sgcd$^{-/-}$ zebrafish. *No statistical differences were observed in length of body (A) and fin (B) between wild type (N=19) and mutated zebrafish (N=24), male and female chosen randomly. On the other hand, the body thickness (C) was statistically significantly smaller in sgcd$^{-/-}$ zebrafish in comparison to age matched wild type animals. Statistical analysis was performed by Mann-Whitney test; n.s, $P > 0.05$; **, $P \leq 0.01$.*

Figure S7

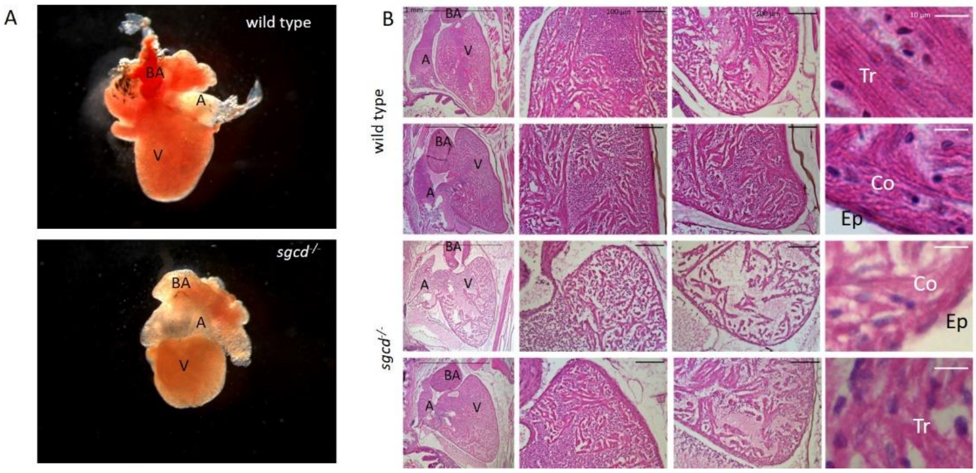

Figure S7. Heart phenotype of adult wild type and sgcd$^{-/-}$ zebrafish. *(A) Representative images of dissected hearts from 1-year-old wild type and sgcd$^{-/-}$ zebrafish. (B) H&E staining of heart sagittal sections from adult wild type and sgcd$^{-/-}$ zebrafish. BA bulbus arteriosus; A, atrium, V, ventricle, Co, compact myocardium; Tr, trabecular myocardium; Ep, epicardium.*

Table S4

Table S4. primary antibodies

ABS	Type	Working concentration	Used for	Company
**ANTI - *SGCD* (N-TERM)**	Rabbit polyclonal	1:200	WB	Antibodies-online (ABIN2781786)
**ANTI - *SGCB* (N-TERM)**	Rabbit polyclonal	1:200	WB	Antibodies-online (ABIN2781786)
**ANTI - DERLIN-1**	Mouse monoclonal	1:2000	WB	Sigma (SAB4200148)
**ANTI - GRP78 (BIP)**	Rabbit polyclonal	1:2000	WB	Abcam (ab21685)
**ANTI - HRD1/SYVN1**	Rabbit polyclonal	1:500	WB	Proteintech (13473-1-AP)
**ANTI - P97 (VCP)**	Rabbit polyclonal	1:1000	WB	Thermo Fisher (PA5-22257)
**ANTI - RNF-5**	Mouse monoclonal	1:500	WB	Santa Cruz Biotechnology (sc-81716)
**ANTI - SEL1L (N-TERMINAL)**	Rabbit polyclonal	1:1000	WB	Sigma (S3699)
**ANTI - BETA TUBULIN**	Rabbit polyclonal	1:2000	WB	Abcam (ab6046)
**ANTI - L-PLASTIN**	Rabbit polyclonal	1:500	IF	Abcam (ab210099)
